# A Pyranose-2-Phosphate Motif Is Responsible for Both Antibiotic Import and Quorum-Sensing Regulation in *Agrobacterium tumefaciens*

**DOI:** 10.1371/journal.ppat.1005071

**Published:** 2015-08-05

**Authors:** Abbas El Sahili, Si-Zhe Li, Julien Lang, Cornelia Virus, Sara Planamente, Mohammed Ahmar, Beatriz G. Guimaraes, Magali Aumont-Nicaise, Armelle Vigouroux, Laurent Soulère, John Reader, Yves Queneau, Denis Faure, Solange Moréra

**Affiliations:** 1 Institute for Integrative Biology of the Cell (I2BC), Department of Biophysics, Biochemistry and Structural Biology, CNRS CEA University Paris-Sud, Gif-sur-Yvette, France; 2 Institute for Integrative Biology of the Cell (I2BC), Department of Microbiology, CNRS CEA University Paris-Sud, Gif-sur-Yvette, France; 3 Institut de Chimie et de Biochimie Moléculaires et Supramoléculaires, ICBMS, Université de Lyon, INSA Lyon, UMR 5246, CNRS, Université Lyon 1, INSA Lyon, CPE-Lyon, Bât J. Verne, Villeurbanne, France; 4 Department of Cell Biology and Physiology, University of North Carolina at Chapel Hill, Chapel Hill, North Carolina, United States of America; 5 Synchrotron SOLEIL, Gif sur Yvette, France; 6 Institute for Integrative Biology of the Cell (I2BC), Protein-Protein Interaction Platform, CNRS CEA University Paris-Sud, Orsay, France; University of California Riverside, UNITED STATES

## Abstract

Periplasmic binding proteins (PBPs) in association with ABC transporters select and import a wide variety of ligands into bacterial cytoplasm. They can also take up toxic molecules, as observed in the case of the phytopathogen *Agrobacterium tumefaciens* strain C58. This organism contains a PBP called AccA that mediates the import of the antibiotic agrocin 84, as well as the opine agrocinopine A that acts as both a nutrient and a signalling molecule for the dissemination of virulence genes through quorum-sensing. Here, we characterized the binding mode of AccA using purified agrocin 84 and synthetic agrocinopine A by X-ray crystallography at very high resolution and performed affinity measurements. Structural and affinity analyses revealed that AccA recognizes an uncommon and specific motif, a pyranose-2-phosphate moiety which is present in both imported molecules *via* the L-arabinopyranose moiety in agrocinopine A and the D-glucopyranose moiety in agrocin 84. We hypothesized that AccA is a gateway allowing the import of any compound possessing a pyranose-2-phosphate motif at one end. This was structurally and functionally confirmed by experiments using four synthetic compounds: agrocinopine 3’-*O*-benzoate, L-arabinose-2-isopropylphosphate, L-arabinose-2-phosphate and D-glucose-2-phosphate. By combining affinity measurements and *in vivo* assays, we demonstrated that both L-arabinose-2-phosphate and D-glucose-2-phosphate, which are the AccF mediated degradation products of agrocinopine A and agrocin 84 respectively, interact with the master transcriptional regulator AccR and activate the quorum-sensing signal synthesis and Ti plasmid transfer in *A*. *tumefaciens* C58. Our findings shed light on the role of agrocinopine and antibiotic agrocin 84 on quorum-sensing regulation in *A*. *tumefaciens* and reveal how the PBP AccA acts as vehicle for the importation of both molecules by means of a key-recognition motif. It also opens future possibilities for the rational design of antibiotic and anti-virulence compounds against *A*. *tumefaciens* or other pathogens possessing similar PBPs.

## Author Summary

We succeeded in understanding how the periplasmic protein AccA from the pathogen *A*. *tumefaciens* can bind both the plant compound agrocinopine and the antibiotic agrocin 84. Whereas agrocinopine acts as a nutrient and regulatory signal in *A*. *tumefaciens*, agrocin 84 is lethal once degraded by the enzyme AccF into a toxic moiety. We identified the pyranose-2-phosphate-like moiety shared by these two ligands as the key recognition template for AccA. We hypothesized that agrocin 84 would kill all agrobacteria possessing AccA and AccF and that AccA would be a gateway allowing the importation of any compound possessing a pyranose-2-phosphate motif. We experimentally confirmed this, using synthetic derivative compounds of agrocinopine. Furthermore, using affinity and *in vivo* assays, we showed that arabinose-2-phosphate, resulting from the cleavage of agrocinopine by AccF, is the effector of the transcriptional repressor AccR, that controls quorum-sensing and virulence plasmid propagation. Therefore, we have identified an original and specific key molecular motif (pyranose-2-phosphate) allowing a selective passage of active compounds into the pathogen cells and acting as signals once the active compounds are cleaved into this key motif. Our work opens up new opportunities to rationally design novel antibiotics.

## Introduction

In bacteria, periplasmic binding proteins (PBPs) are involved in the import into the cell of a wide variety of extracellular compounds. PBPs recognize and bind chemical compounds in order to bring them to ABC transporters which transport them into cells [1]. PBPs are also potential vehicles that facilitate the penetration of antibiotics into bacterial pathogens. To our knowledge, the best known system that exemplifies this paradigm is the antibiotic agrocin 84, which penetrates into the cytoplasm of the bacterial pathogen *Agrobacterium tumefaciens* strain C58 by hijacking the PBP called AccA and its cognate transporter [2,3]. The antibiotic agrocin 84 is produced by the non-pathogenic bacterial strain *Agrobacterium radiobacter* strain K84 [2,3]. Since the 1970’s, *A*. *radiobacter* K84 has been used as a biocontrol agent in several countries to prevent outbreaks of the crown gall disease caused by the pathogen *A*. *tumefaciens* in a wide range of plants [4]. Agrocin 84, having gained access to the *A*. *tumefaciens* C58 cytoplasm, is maturated into a toxic moiety (TM84) that inhibits agrobacterial growth [5,6]. TM84 acts as a tRNA-dependent inhibitor of leucyl-tRNA synthetase that traps the enzyme in a ternary inhibition complex [7] thus preventing tRNALeu aminoacylation and thereby halting protein synthesis.

**Fig 1:**
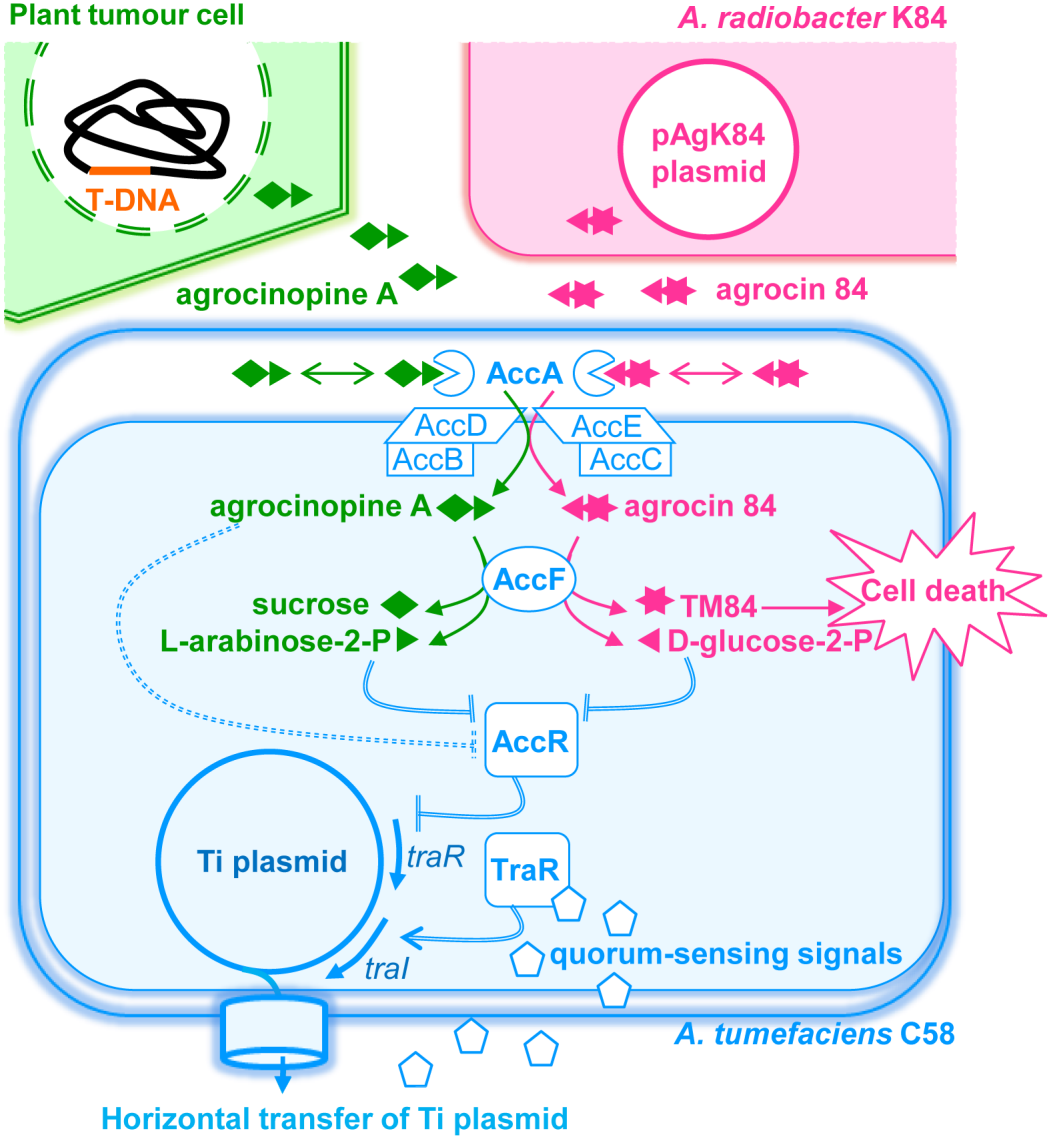
A simplified schematic representing the agrocinopine A and agrocin 84 roles in *A*. *tumefaciens* C58. Upon infection, virulent agrobacteria transfer a small DNA fragment (T-DNA) from its virulence Ti plasmid to the nuclear genome of the plant cells leading to genetically modified plant cells and plant-tumor formation. In plant tumor cells, the bacterial T-DNA encodes the production of opines including agrocinopine A used as nutrients and regulatory signals by agrobacteria which colonize the plant tumour tissues. Agrocinopine A is imported into the bacterial cytoplasm via the periplasmic binding protein AccA associated to a unique ABC-transporter (AccBCDE). Once in the cytoplasm, agrocinopine A is cleaved by the enzyme AccF into sucrose and L-arabinose-2-phosphate. However, Agrocin 84 produced by the non-pathogenic strain *A*. *radiobacter* K84 (which colonizes the same plant environment as the *A*. *tumefaciens* C58 pathogen) uses the same import system AccA and AccBCDE for penetrating into the cytoplasm of *A*. *tumefaciens* C58. AccF cleaves agrocin 84 into D-glucose-2-phosphate and the toxic moiety named TM84 which kills pathogen cells. In *A*. *tumefaciens* C58, agrocinopine A is proposed to interact with the transcriptional repressor AccR (dashed double lines), hence releasing *acc* and *traR* genes expression. Then, the transcriptional activator TraR interacts with quorum-sensing signals and promotes the expression of the *tra*, *trb* and *rep* genes which stimulate the biosynthesis of the quorum-sensing signals (traI), and amplification of copy number and conjugation of the Ti plasmid (regulation steps are indicated by double lines). In our work, we investigated the interactions between AccA and its ligands, as well as those between AccR and L-arabinose-2-phosphate and D-glucose-2-phosphate and their consequence on quorum-sensing and Ti plasmid transfer.

AccA also plays a key-role in the importation of the characteristic plant tumour-derived compounds such as the opines agrocinopines A and B discovered in 1981 in crown gall tumour tissue [8]. Agrocinopine A is composed of a sucrose linked to a L-arabinose via a phosphodiester bond. Agrocinopine B results from the cleavage of the sucrose moiety of agrocinopine A. Hence agrocinopine B is composed of a fructose linked to a L-arabinose via a phosphodiester bond. The agrocinopines A and B were purified from tumours induced by *A*. *tumefaciens* strain C58. Upon infection, *A*. *tumefaciens* genetically engineers the plant host by transferring a piece of DNA (the T-DNA) from its tumour inducing (Ti) plasmid to the nuclear genome of plants. Proliferation of the transformed plant cells results in the formation of tumours colonized by the bacteria. In plant tumours, T-DNA genes expression redirects the metabolism towards the production of several opines which are used by the pathogen as nutrients (C, N and P sources) and signals to control the quorum-sensing signal expression [9–11]. Indeed, agrocinopines A and B play a crucial role in the *A*. *tumefaciens* C58 infection process by inducing the synthesis of the quorum-sensing signal 3-oxo-octanoylhomoserine lactone (OC8HSL) which increases aggressiveness of agrobacterium and activates the dissemination of the Ti plasmid by horizontal transfer (by conjugation) [12,13] (Fig 1). Binding of agrocinopine to the transcriptional repressor AccR, which belongs to the DeoR transcriptional factors family, is proposed to inhibit its repression [14], releasing the expression of a second transcriptional factor TraR, which binds the quorum-sensing signal OC8HSL. In turn, the complex TraR-OC8HSL activates the expression of quorum-sensing regulated genes such as the *tra* and *trb* operons, encoding for horizontal transfer of the Ti plasmid by conjugation and the *rep* operon which controls the replication of the Ti plasmid. In addition to quorum-sensing, AccR also regulates the transcription of *acc* (agrocinopine catabolism) genes [15]. These genes encode an agrocinopine ABC transporter system including the PBP AccA and two putative enzymes required for catabolism of agrocinopine, the phosphodiesterase AccF and the phosphatase AccG.

Agrocin 84 differs from agrocinopine A [16,17,18]. It is composed of a D-glucose-phosphoramidate linked to TM84. The phospho-glucose moiety of agrocin 84 is required for its import into pathogenic *A*. *tumefaciens* cells [6]. Agrobacterium phosphodiesterase AccF should cleave the antibiotic agrocin 84 into a D-glucose-phosphate and TM84, whereas the same enzyme cleaves agrocinopine A into L-arabinose-2-phosphate and sucrose. Using X-ray crystallography, we investigated at the atomic level the binding mode of AccA with the antibiotic agrocin 84 and the virulence signal agrocinopine A, which are chemically different and exhibit antagonist functions. Agrocin 84 was purified from an agrobacterial strain containing a derivative of the plasmid pAgK84 that allows agrocin 84 overproduction from cells [18], while agrocinopine A was synthetized through a multistep protocol. Based on the structural analysis of AccA structures in complex with agrocinopine A or agrocin 84, we then focussed on three additional potential ligands which are analogues of agrocinopine A: agrocinopine 3’-*O*-benzoate, L-arabinose-2-phosphate and L-arabinose-2-isopropylphosphate. To address the structural basis for AccA specificity, the carbohydrate fragment of agrocin 84, namely D-glucose-2-phosphate was also synthetized. To understand the regulation mode of the AccR transcriptional repressor activity and the role of the opine agrocinopine A and its AccF-mediated degradation product arabinose-2-phosphate, we combined affinity measurements and a plasmid conjugation assay using the wild-type strain and a defective strain mutant *accF* of *A*. *tumefaciens* C58. We found that agrocinopine A is not the effector of AccR as previously described [14], in contrast to L-arabinose-2-phosphate and D-glucose-2-phosphate which both activate the quorum-sensing signal synthesis and the plasmid Ti conjugation. Because L-arabinose-2-phosphate (from agrocinopine A) and D-glucose-2-phosphate (from agrocin 84) are compounds which are unique among the natural products described so far, this work reveals that *A*. *tumefaciens* evolves a pyranose-2-phosphate motif which is the key-recognition pattern by the PBP AccA for bacterial importation and by the master transcriptional regulator AccR for quorum-sensing activation and *acc* operon expression.

## Results

### Synthesis of agrocinopine A, its derivatives and D-glucose-2-phosphate

**Fig 2:**
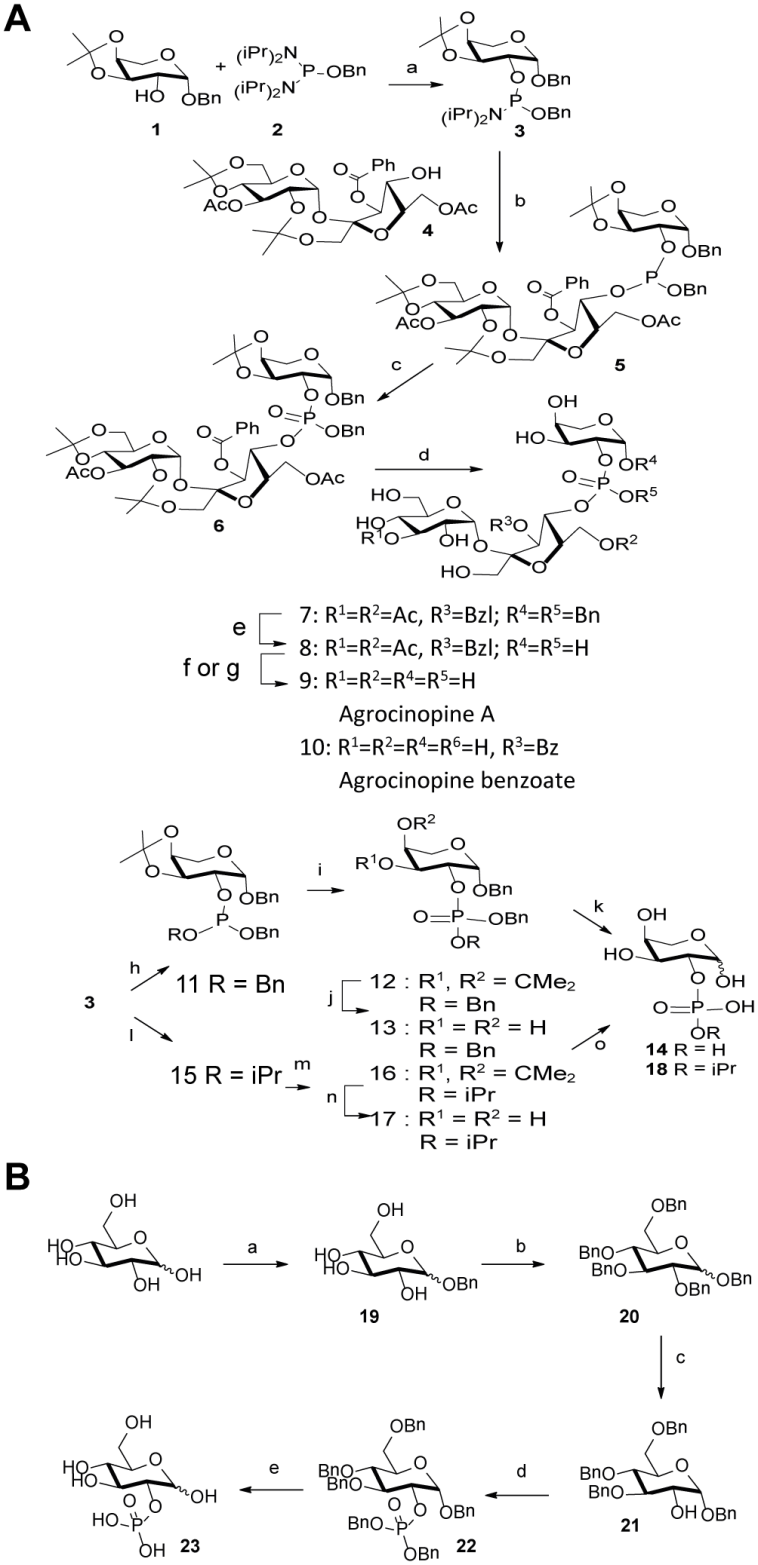
Synthesis scheme. (A) agrocinopine A and its derivatives. Reagents and conditions: (a) 1H-tetrazole, diisopropylamine, CH2Cl2, 2 h, 79% (b) 1H-tetrazole, CH2Cl2, 2 h, 64% (c) tBuOOH, octane, CH2Cl2, 2 h, 92%; (d) 60% aqueous acetic acid, 50°C, 30 min, 53% (e) H2, Pd/C,1 atm, 24 h, 83%(f) 1M methanolic MeONa, methanol, 30 min, 9+10 (g) K2CO3, methanol, 2h, 9, 34–68% (h) BnOH, 1H-tetrazole, CH2Cl2, 30 min, 84% (i) tBuOOH, octane, CH2Cl2, 30 min, 93%; (i) 60% aqueous acetic acid, 50°C, 30 min, 69% (k) H2, Pd/C, 1 atm, 24 h, quant. (l) isopropanol, 1H-tetrazole, CH3CN, 1 h, 50% (m) tBuOOH, octane, CH2Cl2, 30 min, quant. (n). 60% aqueous acetic acid, 50°C, 30 min, 64% (o) H2, Pd/C, 1 atm, 24 h, quant. (B) D-glucose-2-phosphate. Reagents and conditions: (a) BnOH, sulfamic acid, 80°C, neat, 10h, 22% (α/β = 5:2); (b) BnBr, NaH, DMF, rt, 18h, 86%; (c) TIBAL, toluene, 50°C, 60h, 26% (100% α); (d) 1H-tetrazole, (BnO)2-P-N(iPr)2, CH2Cl2, 2h, then m-CPBA, 0°C to rt, 2h, 84%; (e) H2, Pd/C, methanol, 18h, 87%.

The synthesis strategy for the production of agrocinopine A (Fig 2) allowed us to produce derivatives, which are L-arabinose-2-phosphate, L-arabinose-2-isopropylphosphate and agrocinopine-3’-*O*-benzoate (S1 Fig). A D-glucose-2-phosphate was also synthetized (Fig 2 and S1 Fig).

### AccA fold is a PBP from Class C

**Fig 3:**
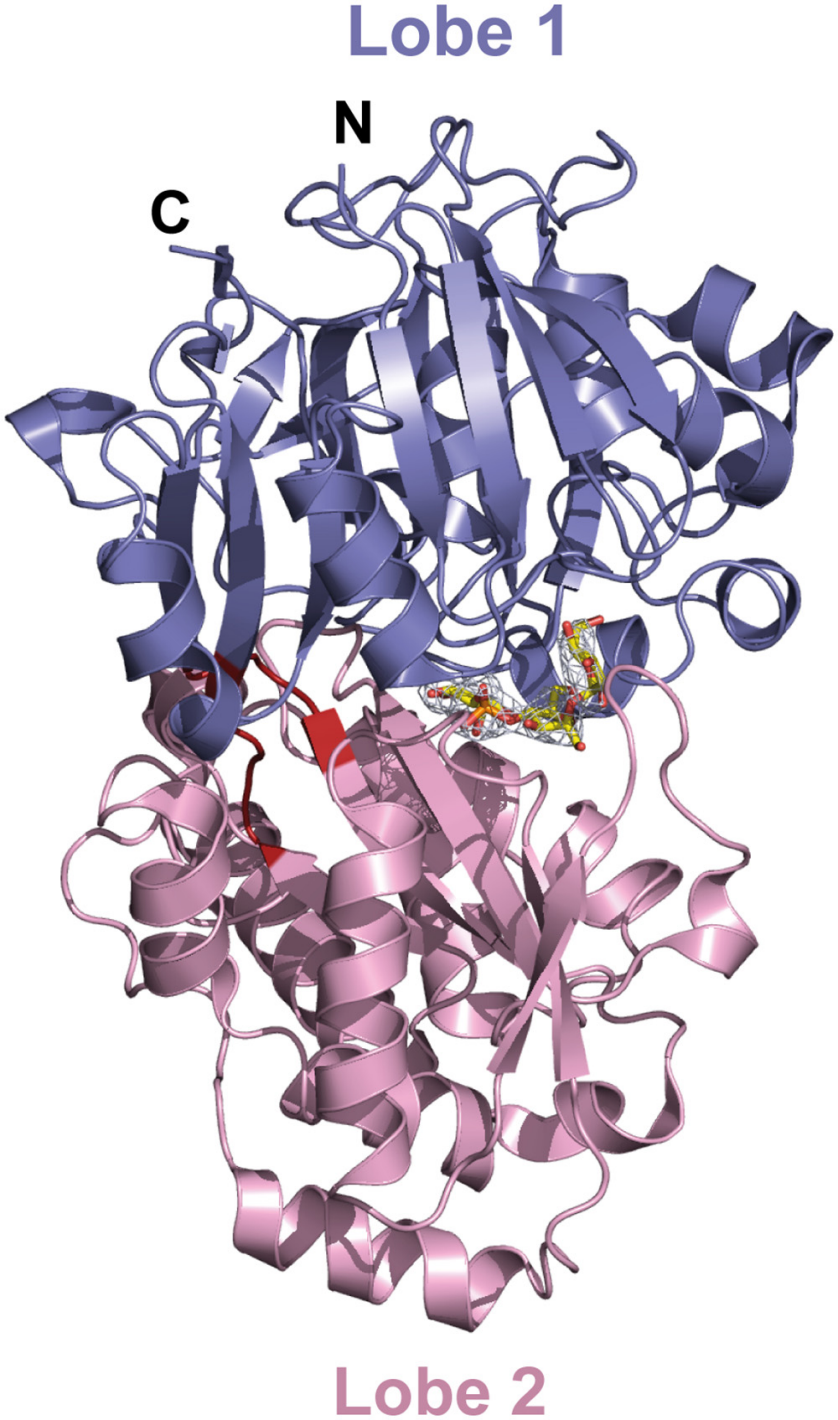
Ribbon representation of AccA in complex with agrocinopine A shown in its annealing Fo-Fc omit map contoured at 4δ. Agrocinopine A is located in the cleft between the lobe 1 (residues 29–280 and 494–521) in slate and the lobe 2 (residues 285–489) in pink and is represented as yellow stick. The short hinge region is shown in red.

The mature AccA expression plasmid was obtained by cloning the *accA* gene lacking the first twenty-nine signal sequence residues that serve for localization to bacterial periplasm. Because AccA shares low sequence identity (around 20%) compared to PBPs with known three dimensional structures, we first solved the structure of seleniated AccA in complex with agrocinopine A at 2.65 Å resolution by single-wavelength anomalous dispersion method. A better resolution structure of this complex at 1.9 Å resolution in a different space group using the native protein was determined. The two agrocinopine-complexed structures are very similar, displaying an average root mean square deviation (rmsd) of 0.35 Å over 489 Cα atoms. By the molecular replacement method, we then solved the structure of AccA in complex with agrocin 84 at 2.15 Å resolution (Table 1). The mature AccA structure is a monomer of 493 residues composed of two lobes, each formed by a central β-sheet flanked by α-helices. The biggest lobe (lobe 1) consists of residues 29–280 and 494–521 and the smallest (lobe 2) comprises the residues 285–489. The two lobes are connected by a very short hinge region of 8 residues defining two short segments (Fig 3). AccA fold belongs to the cluster C within the PBP structural classification [1]. Overall, the ligand bound structures are very similar (average rmsd value of 0.33 Å over 490 Cα atoms) adopting a closed conformation. The major difference concerns the region comprising the residues 403–408, which can move up to 1.7 Å to accommodate the ligand in the binding site.

**Table 1:** Crystallographic data and refinement parameters.

AccA PDB code	4ZE8	4ZE9	4ZEB	4ZED	4ZEC	4ZEI	4ZEK	4RA1
Ligand	No	Agrocinopine-SeMet-	Agrocinopine-	Agrocinopine benzoate	Agrocin 84	L-Arabinose-2-phosphate	L-Arabinose-2-isopropylphosphate	D-Glucose-2-phosphate
Crystallization conditions	A: 25% P8K, 0.1M SA, 5% glycerol, acetate Na pH 4.5	B: 20% P4K, 0.2M acetate NH4, 0.1M citrate Na pH 5.6	C: 15% P20K, 0.1M MES pH 6.5	B	B	B	B	B
**Data collection** ^a^								
Space group	P2_1_	C222_1_	C2	C222_1_	C222_1_	C222_1_	C222_1_	C222_1_
a/b/c (Å)	72.9/179.3/81.6	77.7/114.7/109.1	177.2/51.2/120	77.6/114.9/113.9	77.2/114.2/111.7	78/114.9/109.8	77.2/114.1/112.5	77.2/113.8/113.5
α/β/γ (°)	90/93.5/90	90/90/90	90/114.3/90	90/90/90	90/90/90	90/90/90	90/90/90	90/90/90
mol/UA	4	1	2	1	1	1	1	1
Resolution (Å)	45–1.71	41.59–2.65	50–1.9	50–1.75	45–2.15	50–2.3	42.2–2.10	50–1.75
	(1.82–1.71)	(2.81–2.65)	(2.01–1.89)	(1.85–1.75)	(2.28–2.15)	(2.44–2.3)	(2.22–2.10)	(1.85–1.75)
Total reflections	729622 (79336)	154373 (20743)	538420 (82654)	590716 (86690)	163437 (22778)	97607 (15267)	168570 (25685)	329935 (47160)
Unique reflections	214418 (29651)	14551 (2281)	78059 (9195)	51499 (7828)	26755 (4191)	22254 (3489)	29355 (4552)	50794 (7954)
Redundancy	3.4	10.6	6.9	11.5	6	4.4	5.7	6.5
Completeness (%)	96.7 (82.7)	99.8 (98.9)	98.8 (95.8)	99.2 (96.9)	98.0 (96.7)	99.6 (98.5)	99.4 (96.6)	99.6 (97.7)
I/σi (*I*)	8.9 (1.7)	11.78 (2.23)	7.87 (1.26)	12.25 (1.81)	8.25 (1.27)	8.48 (1.20)	7.88 (1.36)	12.29 (1.71)
CC_1/2_ ^b^	99.6 (79.4)	99.6 (73.7)	99.4 (54.2)	99.2 (67.3)	99.8 (71.2)	99.4 (59.0)	99.4 (59.6)	99.8 (81.8)
Rsym (%)	9.4 (54.3)	11.9 (64.2)	11.1 (92.6)	9.2 (84.4)	21.2 (104)	14.6 (117.9)	18.2 (130.4)	8.9 (102.0)
**Refinement**								
R factor/ R free (%)	16.6/ 19.7	17.1/23.2	17.2/20	17.4/19	19.8/23.2	20/24.9	18.7/23.7	17.6/20.2
Rmsd bond (Å) / angle°	0.01/1.03	0.008/ 1.05	0.01/1.06	0.009/1.01	0.008/ 1.13	0.009/1.11	0.009/1.09	0.010/1.03
Mean B factor (Å2)								
protein	23.3	43.7	36.5	28.9	36.3	50.6	39.9	33.44
solvent	33.8	42	39.8	35.3	40.7	52.28	39.1	39.02
ligand		39.6	35.65	34.9	50.8	42.40	35.8	23.26

^a)^ Values in parenthesis are those for the last shell.

^b)^ CC_1/2_ = percentage of correlation between intensities from random half‐dataset (P. A. Karplus, K. Diederichs, Science 2012, 336, 1030–1033).

The unliganded AccA structure solved at 1.7 Å resolution adopts an open conformation. Indeed, superposition with the liganded structures results in an average rmsd of 1.5 Å for all Cα atoms and structural comparisons show that upon ligand binding, a 12° rotation around an axis defined by the two hinge residues, Met292 and Tyr493, leads to a similar closed form of the protein.

### AccA binds a part of agrocinopine A and agrocin 84 ligands

Both ligands are bound between the two closed lobes of AccA. While Agrocinopine A is fully defined in the electron density maps, the leucine-like moiety of agrocin 84 is mobile (S1 Fig). Notably, the electron density maps of agrocin 84 show a D-glucopyranose connected via its C2 carbon to the N6-phosphoramidated TM84 moiety and not a D-glucofuranose connected via a C1-linkage as previously suggested using analytical chemistry approaches that were available at the end of the 1970’s [2,3,19]. Later, a possible D-glucopyranose-C2 structure for the uptake moiety of agrocin 84 has been proposed [20,21].

**Fig 4:**
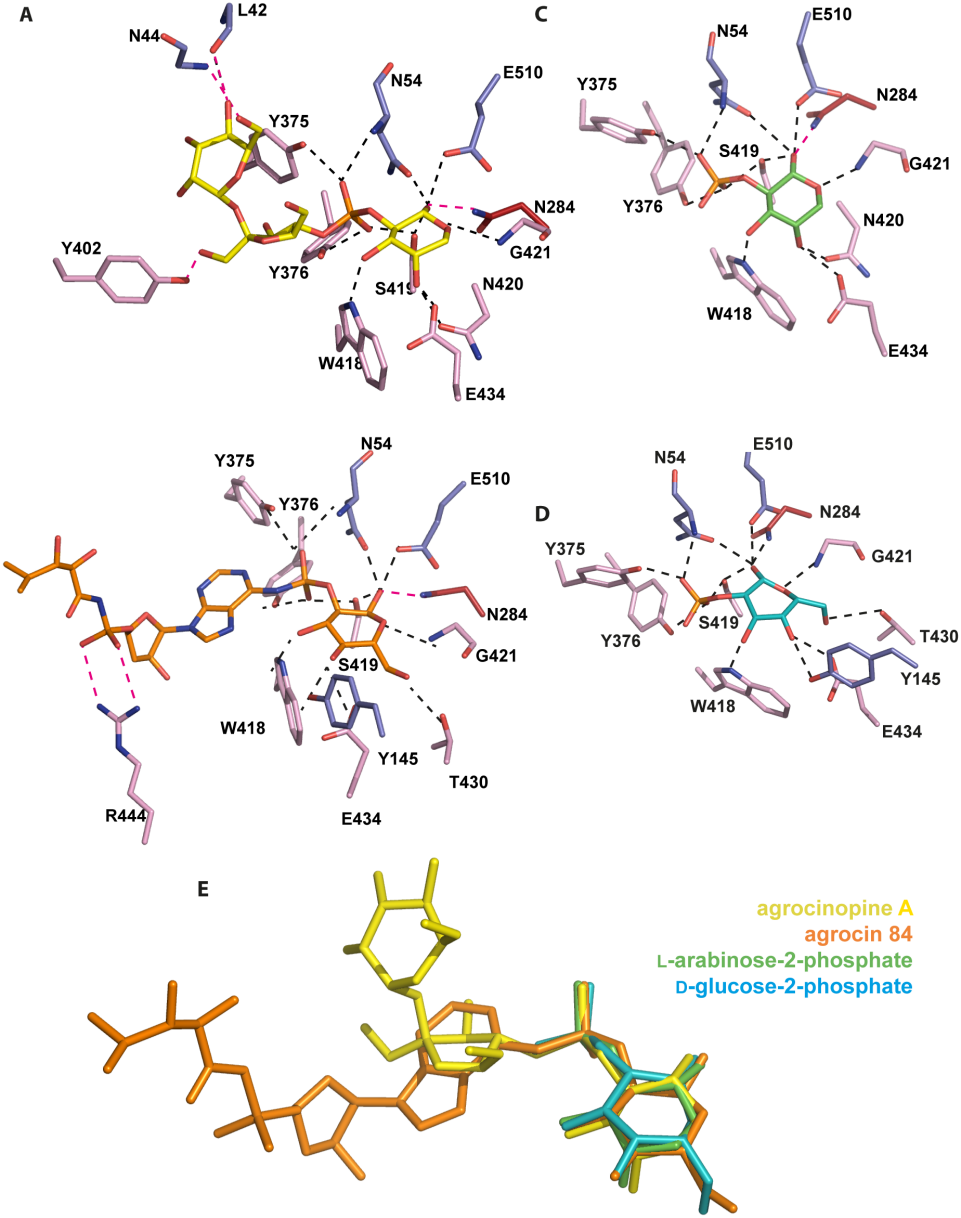
Ligand-binding site of AccA. Ligands and protein residues involved in the ligand binding are shown as stick. Hydrogen bonds are shown as dashed lines in black (for distances below 3.2 Å) and magenta (for distances between 3.2 and 3.4 Å). (A) agrocinopine A. (B) agrocin 84. (C) L-arabinose-2-phosphate. (D) D-glucose-2-phosphate. (E) Superimposition of the four ligands bound in the ligand binding site of AccA, agrocinopine A (yellow), agrocin 84 (orange), L-arabinose-2-phosphate (green) and D-glucose-2-phosphate (cyan) are shown as stick.

Both bound ligands, agrocinopine A and agrocin 84, share a deeply buried pyranose (L-arabinose or D-glucose)-2-phosphate-like moiety which superimposes very well in the AccA binding site, and makes numerous and very similar protein contacts (Fig 4). The pyranose-2-phosphate moiety lies on residues 418–421 from the strand β16 of lobe 2 and is surrounded by the N-terminal loop region 52–54 and the side chains of Tyr145, Trp178, Glu504 and Glu510 from lobe 1, that of Asn284 from the hinge region and those of Met372, Tyr375, Tyr376, Thr430, Glu434 from lobe 2. It makes extensive protein hydrogen bonds: 8 for L-arabinose and 10 for D-glucose (Fig 4A and 4B). Remarkably, the OH1 group of the pyranose is anchored by 4 hydrogen bonds involving Asn54 and Glu510 from lobe 1, Asn284 from the hinge region and Ser419 from lobe 2. These four side chains form a rigid template, which also maintains the lobe closure by interacting together two-by-two (S2a Fig). Moreover, in addition to their pyranose interactions, two of these major residues Asn54 and Ser419 also tightly interact with the phosphate/phosphoramidate groups. Two more hydrogen bonds involving the side chains of two tyrosines 375 and 376 retain the phosphate/phosphoramidate oxygens. In contrast, the sucrose moiety of agrocinopine A and the TM84 part of agrocin 84 make only 3 and 2 polar interactions with AccA, respectively. The sucrose moiety and TM84 occupy a different position in the binding site.

### AccA recognizes the pyranose-2-phosphate-like motif

We determined the structures of AccA in complex with several synthetic derivatives of agrocinopine A and agrocin 84: L-arabinose-2-isopropylphosphate, L-arabinose-2-phosphate and D-glucose-2-phosphate at 2.1, 2.3 and 1.75 Å resolution respectively (Table 1). L-arabinose relatives and D-glucose-2-phosphate are well defined in the electron density maps (S1 Fig). The L-arabinose-2-isopropylphosphate and L-arabinose-2-phosphate alone or within agrocinopine A make the same protein interactions and superimpose well (Fig 4C and 4E). A similar observation can be made for the bound D-glucose-2-phosphate (Fig 4D and 4E). To summarize, AccA binds the pyranose-2-phosphate/phosphoramidate key-template through numerous polar interactions and its selectivity for this motif was validated by the structures of AccA in complex with L-arabinopyranose-2-phosphate and D-glucopyranose-2-phosphate.

### Binding and assimilation of a synthetic bulky ligand containing the minimal pyranose-2-phosphate motif

The synthesis process of agrocinopine A allows obtaining bulky agrocinopine A derivatives such as agrocinopine 3’-*O*-benzoate. We used this compound to test whether AccA could bind a synthetic ligand exhibiting the minimal pyranose-2-phosphate motif. We therefore co-crystallized AccA with this compound. The structure at high resolution shows a very well defined bound agrocinopine 3’-*O*-benzoate (S1 Fig). Interestingly, while the L-arabinose-2-phosphate between bound agrocinopine 3’-*O*-benzoate and agrocinopine A superimpose, their sucrose moieties do not occupy the same position. Whereas the benzoate group of the agrocinopine 3’-*O*-benzoate adopts the position of the sucrose in agrocinopine, its sucrose moiety follows that of the TM84 (S2b Fig).

When present as the sole carbon source for *A*. *tumefaciens* C58, agrocinopine 3’-*O*-benzoate is used as nutrient by the bacteria as indicated by bacterial growth (S3 Fig). A similar behaviour is observed with L-arabinose-2-phosphate used as a sole carbon source meaning that agrocinopine 3’-*O*-benzoate is degraded in the bacterial cytoplasm once imported by AccA and its ABC transporter. This experiment confirmed that the transporter can uptake bulky synthetic molecules such as agrocinopine 3’-*O*-benzoate into the cell as observed for the toxin agrocin 84, which is also a relatively large molecule.

### AccA exhibits a high affinity for the pyranose-2-phosphate-like motif

**Fig 5:**
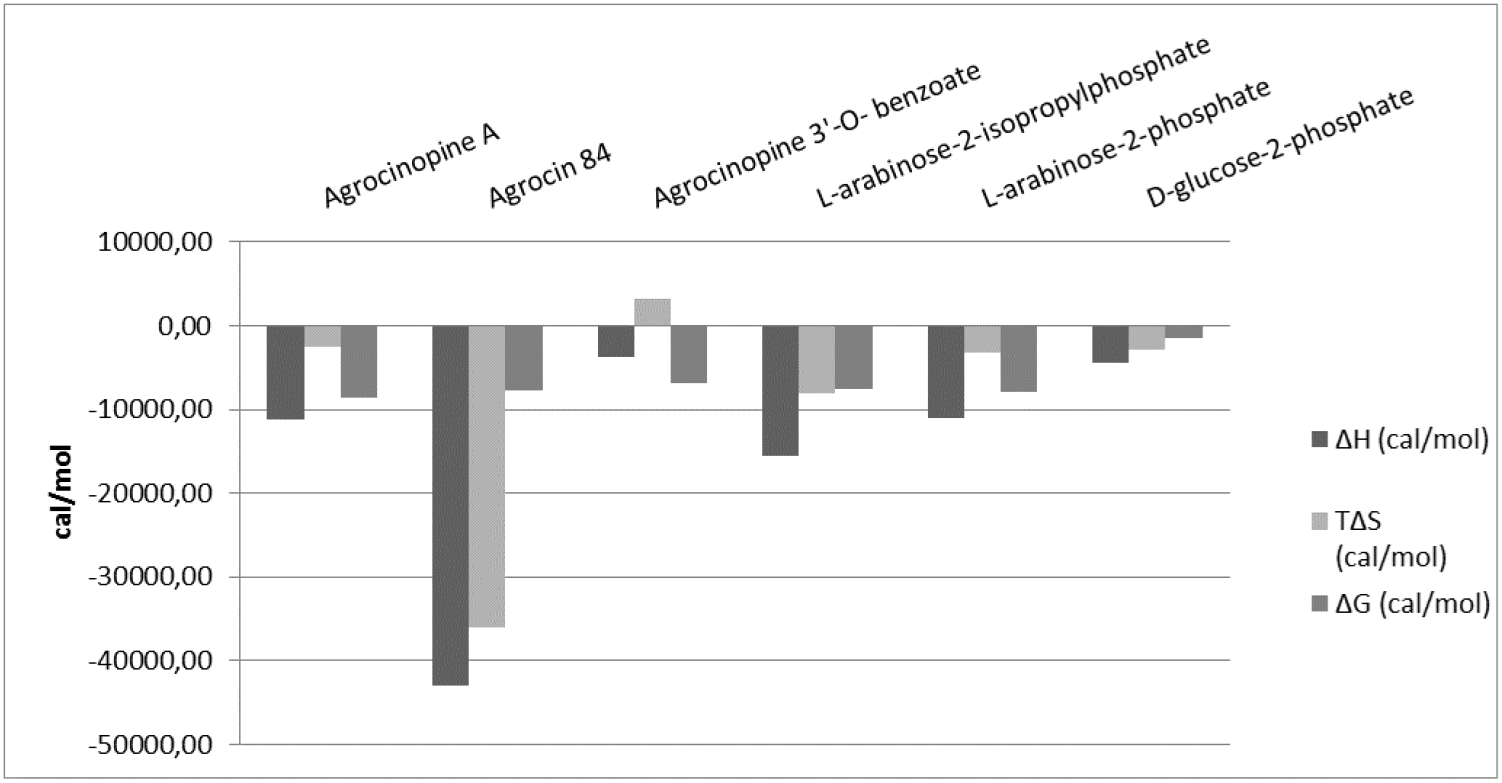
Comparison of microcalorimetry derived enthalpy (ΔH, deep grey), entropic contribution (TΔS, grey) and free binding enthalpy (ΔG, light grey) at 293°K for agrocinopine A, agrocin 84, agrocinopine 3’-*O*-benzoate, L-arabinose-2-isopropylphosphate, L-arabinose-2-phosphate and D-glucose-2-phosphate.

Ligand binding to the protein AccA was investigated using tryptophan fluorescence spectroscopy, a method exploiting significant conformational changes accompanying the binding. The autofluorescence intensity enhancement correlated with the ligand concentrations between 0.3 and 20 μM and saturated above 50 μM. Titration experiments yielded apparent *K*_*D*_ values of 1.3 ± 0.17 μM, 5.88 ± 1.6 μM, 2.93 ± 0.66 μM, 4.79 ± 0.6 μM and 2.5 ± 0.5 μM with agrocinopine A, agrocinopine 3’-*O*-benzoate, L-arabinose-2-phosphate, L-arabinose-2-isopropylphosphate and D-glucose-2-phosphate, respectively (S4 Fig and S1 Table). No fluorescence intensity change was detected by incubating AccA with L-arabinose, D-glucose or phosphate alone. As expected from models based on the X-ray liganded AccA structures, no autofluorescence signal changes were measured by incubating AccA with glucose-1-phosphate or glucose-6-phosphate, which both are common metabolites in all living organisms. Unexpectedly, no signal was measured with agrocin 84, likely due to the presence of the adenosine moiety of agrocin 84 which provokes a quenching signal. Therefore, we used isothermal titration microcalorimetry to assess the binding of agrocin 84 to AccA, which yielded a mean *K*_*D*_ of 1.5 ± 0.41 μM. The mean *K*_*D*_ values were 0.3 ± 0.03 μM, 7.5 ± 2.2 μM, 1.33 ± 0.12 μM, 2.2 ± 0.58 μM and 1.16 ± 0.22 μM for agrocinopine A, agrocinopine 3’-*O*-benzoate, L-arabinose-2-phosphate, L-arabinose-2-isopropylphosphate and D-glucose-2-phosphate respectively, consistent with the values obtained from fluorescence spectroscopy. The isothermal titration microcalorimetry data also confirmed the 1:1 binding stoichiometry and demonstrate a negative enthalpy change upon each ligand binding (S5 Fig and S2 Table), suggesting that the binding is enthalpy driven. The similar binding isotherms for all ligands suggest a same binding mechanism involving polar interactions, in agreement with what is observed in the complexed structures (Fig 5). Nevertheless, the benzoate group of the agrocinopine 3’-*O*-benzoate molecule appears to be responsible for an entropic effect leading to a 25-fold lower affinity of this ligand with AccA in comparison with agrocinopine A. We could not detect any binding of L-arabinose, D-glucose, nor adenosine monophosphate in line with the results obtained by fluorimetry.

### Structural comparison between AccA and other PBPs

A structural comparison of AccA with all PDB entries using SSM-EBI (http://www.ebi.ac.uk/msd-srv/ssm) [22] indicates that the most similar overall structures are PBPs from the same cluster: the structure of *Staphylococcus aureus* NikA in complex with nickel and histidine (PDB: 4XKN) [23], *Bacillus subtilis* AppA with a bound nanopeptide (PDB: 1XOC) [24], *Escherichia coli* OppA with different bound tripeptides (PDB: 1JET, 3TCG, 1B46, 3TCF) [25–27], *Escherichia coli* NikA with a bound nickel and histidines (PDB: 4I8C) [28], *Thermatoga maritima* CBP (tmCBP) with bound cellobiose and cellopentaose (PDB: 2O7I and 3IO5) [29] and *Escherichia coli* DPP with a bound dipeptide (PDB: 1DPP) [30]. The values of rmsd range from 1.9 Å to 2.7 Å over 413 to 434 residues. Superposition with other PDB-entries results in an rmsd of over 2.7 Å for less than 410 Cα atoms. Therefore, the most similar PBPs bind either oligopeptides or nickel ions or oligosaccharides. The ligand binding site of these PBPs lies on a conserved β-strand (β14 in AccA) except that of tmCBP. A particularity of AccA which is not shared by these related PBPs is the presence of the flexible loop 402–414 located between the β-strands β15 and β16 which can accommodate agrocinopine A. This loop corresponds to a conserved rigid helix in all similar PBPs and models superposition shows that an equivalent helix in our structure would clash with the last glucose of agrocinopine A (S6a Fig). Therefore the flexible loop 402–414 in AccA seems so far unique among PBPs from cluster C.

The comparison with the oligopeptide binding proteins reveals that AccA resembles the *E*. *coli* DPP, which can bind short oligopeptides (dipeptides) only due to the presence of a loop (residues 344–350) which creates a steric hindrance and reduces the size of the ligand binding site on one side. The position of the dipeptide ligand in DPP closely matches the position of the L-arabinose-phosphate group in AccA despite the large differences in their binding mode. Interestingly, two tryptophan side chains (W178 and W423) from each AccA lobe, which occupy equivalent positions of Met152 and Asp408 in DPP form a gate that closes the ligand binding site preventing the binding of longer substrates linked beyond the pyranose moiety on the O6 or O5 atoms (S6b Fig).

Structural comparison between AccA and NikA binding sites shows that the two bound histidines in NikA overlap the L-arabinose-2-phosphate moiety in AccA with the nickel ion overlapping the oxygen atom linking the arabinose to the phosphate. However, the loop region 51–55 in AccA prevents the interaction of a histidine in the binding site of AccA. An equivalent loop in tmCBP would restrain its ligand binding site preventing the binding of long oligosaccharides. TmCBP binds oligosaccharides ranging from two rings (cellobiose) to five (cellopentaose) in a deep groove at the domain interface. The minimal cellobiose binding site is found at one extreme of this groove and provides extensive network of hydrogen bonds for the di-saccharide. In contrast, the cellopentaose binding site spans the entire interface in a large cavity lacking aromatic and polar residues for the last three sugars. Therefore, tmCBP exhibits specificity only for the cellobiose moiety of the cellopentaose. Although the position and conformation of the binding site between tmCBP and AccA differ (S7 Fig), both PBPs select a molecule class rather than single species.

### L-arabinose-2-phosphate and D-glucose-2-phosphate are effectors of AccR

**Fig 6:**
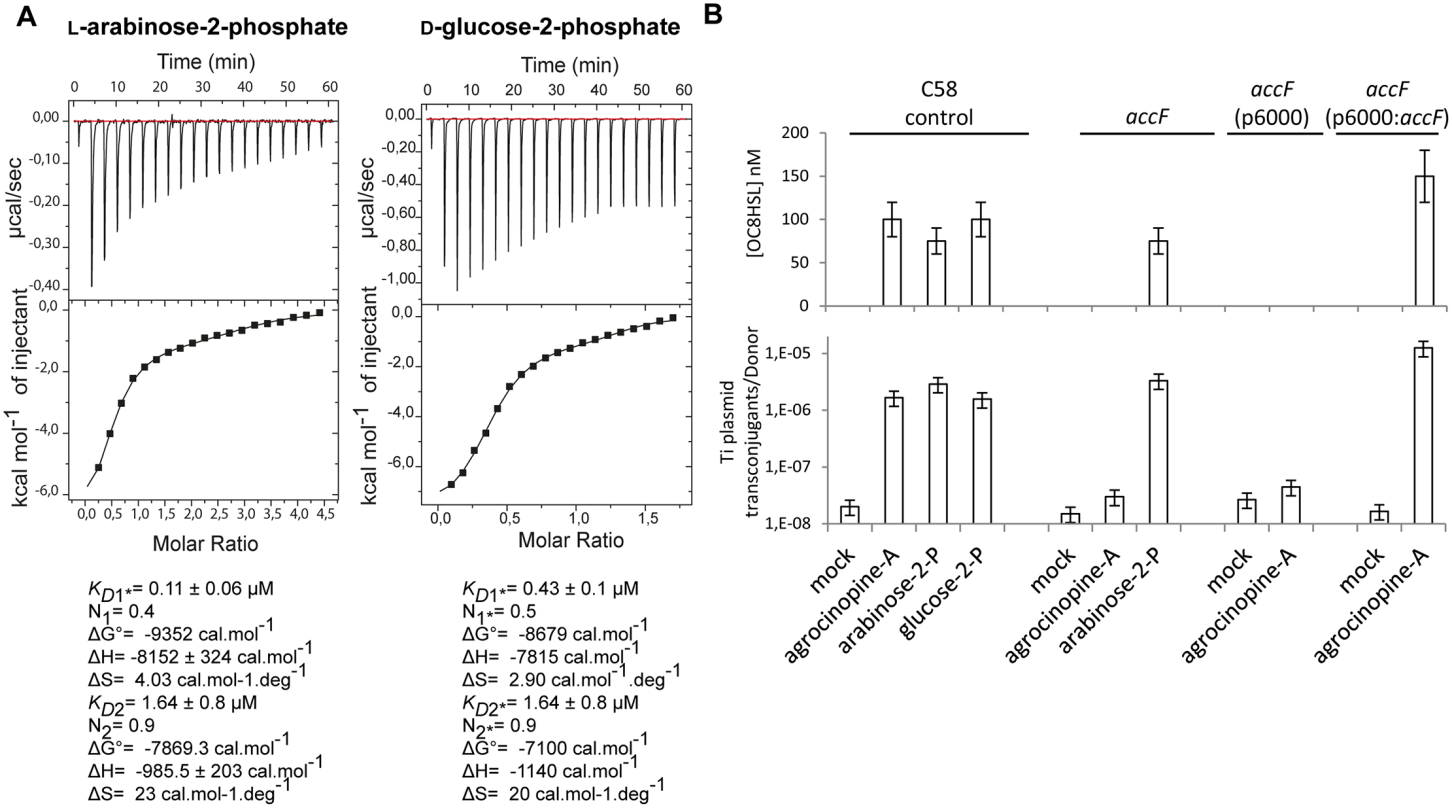
**(A)** AccR microcalorimetry measurements. The top panels show heat differences upon injection of ligand (L-arabinose-2-phosphate on the left and D-glucose-2-phosphate on the right) and lower panels show integrated heats of injection and the best fit (solid line) using Microcal Origin. Fitting values are indicated below. (B) Quantification of OC8HSL production and Ti plasmid conjugation induced by agrocinopine A, L-arabinose-2-phosphate and D-glucose-2-phosphate. Different *A*. *tumefaciens* C58 backgrounds were used as donor cells: C58 control, *accF* mutant, *accF* mutant complemented with p6000: *accF* wild type and *accF* mutant complemented with empty p6000). Results were obtained after 72 h of culture. SDs correspond to physical replicates. Experiments independently repeated between two and four times produce similar results.

In the *A*. *tumefaciens* C58 cytoplasm, the enzyme AccF releases the L-arabinose-2-phosphate and D-glucose-2-phosphate from agrocinopine A and agrocin 84. The question arose about their biological role in *A*. *tumefaciens*, especially in relation to regulation of the AccR/quorum-sensing pathway that controls dissemination of the Ti plasmid. We first measured the affinity of the synthetic agrocinopine A towards purified recombinant AccR by isothermal titration microcalorimetry. An unexpected outcome was that no affinity was detected. In contrast, the isothermal titration microcalorimetry data show that AccR displays affinity to L-arabinose-2-phosphate and D-glucose-2-phosphate in two steps with *K*_*D*_ values for L-arabinose-2-phosphate of *K*D1 = 0.11 ± 0.06 (n1 = 0.4) μM for the first step and *K*D2 = 1.64 ± 0.8 (n2 = 0.9) μM for the second step and for D-glucose-2-phosphate *K*D1* = 0.43 ± 0.1 (n1* = 0.5) μM and *K*D2* = 1.64 ± 0.8 μM (n2* = 0.9) (Fig 6A). The binding stoichiometries of 2:1 (protein:ligand) in the first step and 1:1 in the second step are in line with the fact that AccR forms a dimer in solution as observed by native gel electrophoresis, suggesting that the binding of the effector to AccR may be described as following: first, a ligand binds to one molecule within the dimer before another binds to the second molecule. These results suggest L-arabinose-2-phosphate and D-glucose-2-phosphate are the effector molecules that regulate AccR and thus they should activate quorum-sensing synthesis in *A*. *tumefaciens* cells.

### L-arabinose-2-phosphate and D-glucose-2-phosphate activate the quorum-sensing and plasmid Ti transfer in *A*. *tumefaciens* C58

*In vivo*, the functions of agrocinopine A, L-arabinose-2-phosphate and D-glucose-2-phosphate as quorum-sensing effectors were examined by measuring the production of quorum-sensing molecules and efficiency of Ti plasmid transfer in conjugation between *A*. *tumefaciens* donors and the recipient *A*. *tumefaciens* C58.00 which is cured of plasmids (Fig 6B). Results indicated that all cell cultures supplemented with agrocinopine A, L-arabinose-2-phosphate and D-glucose-2-phosphate accumulate high concentrations of quorum-sensing molecules (between 50 and 100 nM) compared with mock cultures (less than 1 nM). Consistently, Ti plasmid transconjugants could also be detected in the supplemented cultures, demonstrating that agrocinopine A, L-arabinose-2-phosphate and D-glucose-2-phosphate are activators of the quorum-sensing pathway of *A*. *tumefaciens* C58.

Finally, because the purified AccR exhibited affinity for L-arabinose-2-phosphate and not for agrocinopine A *in vitro* and because L-arabinose-2-phosphate activated quorum-sensing and plasmid Ti conjugation *in vivo*, we tested the hypothesis of the key-role of AccF in the maturation of agrocinopine A into L-arabinose-2-phosphate as the efficient activator of quorum-sensing signal. We used an *accF* defective mutant [14]. Remarkably, both accumulation of quorum-sensing molecules and Ti plasmid conjugation were abolished in the *accF* mutant supplemented with agrocinopine A, but restored by genetic complementation of *accF* demonstrating the key role of AccF in quorum-sensing signaling (Fig 6B). Moreover, quorum-sensing signals were still observed in the presence of L-arabinose-2-phosphate in both wild type and *accF* mutant (Fig 6B). Altogether, our findings prove that agrocinopine A needs to be degraded into L-arabinose-2-phosphate, whose interaction with the master regulator AccR induces the quorum-sensing regulation of the conjugative transfer of the Ti plasmid in *A*. *tumefaciens* C58 cells. The pyranose-2-phosphate motif with L-arabinose-2-phosphate and D-glucose-2-phosphate are the effectors of AccR.

## Discussion

Understanding the molecular mechanism whereby toxic or beneficial compounds are imported into bacteria is a major issue, whether it aims at to understanding their biological and ecological roles or to design antibiotics. In this work, we characterized the binding mode of agrocinopine A and agrocin 84 in the PBP AccA thanks to synthetic agrocinopine A and to agrocin 84 purified from an agrobacterial strain containing a derivative of the pAgK84 plasmid. Our structural work showed that the toxin agrocin 84, composed of a glucose moiety linked at its position 2 to TM84, is recognized through a D-glucopyranose-moiety, whereas only a furanose linked at position 1 form had been previously suggested for the structure of agrocin 84 [19]. Our structural analysis of AccA ligand binding site combined with spectrofluorescence and microcalorimetry studies reveal that AccA recognizes, through numerous polar interactions, only the pyranose-2-phosphate/phosphoramidate motif of agrocinopine A, agrocin 84 and other relatives such as agrocinopine 3’-*O*-benzoate in which the sugar is either a D-glucopyranose or a L-arabinopyranose. AccA cannot recognize a phosphate alone, nor a D-glucose, nor a L-arabinose, nor a glucose linked to a phosphate via an oxygen atom other than its O2. The selectivity of AccA for this ligand motif was validated by the structures of AccA in complex with L-arabinose-2-phosphate and D-glucose-2-phosphate and their affinity measurement. Indeed, the *K*_*D*_ for L-arabinose-2-phosphate is very similar to those of longer/bulkier ligands having this group, such as agrocinopine A, agrocinopine 3’-*O*-benzoate, and L-arabinose-2-isopropyl phosphate. Hence, structural and affinity data on AccA with different ligands revealed that AccA is sufficiently flexible to accommodate bulkier ligands as long as they possess the key motif on one end. Moreover, given that the extended ligand agrocin 84 is imported into the pathogen [15,31] in addition to the bulky agrocinopine 3’-*O*-benzoate shown in this study, and since Tate and Kerr [32] have shown that a derivative compound of agrocin 84 lacking the leucine-like portion is transported into the bacteria cell, the transporter associated with AccA does not restrict the size or length of the transported molecules. Thus, it should import any natural or synthetic molecules at least as bulky as the agrocin 84 and agrocinopine 3’-*O*-benzoate that could be identified or designed for improving control of the *A*. *tumefaciens* pathogen. The example of tmCBP [29], which also exhibits limited specificity for the first two sugar rings from its carbohydrate ligand emphasizes that PBPs have promising properties that can be exploited by both natural and synthetic antibiotics that employ a “Trojan Horse” strategy.

Arabinose-2-phosphate and glucose-2-phosphate are uncommon and original molecules in the living world due to the unusual phosphate linkage on the C2 atom of the pyranose. No atomic structure of these molecules has been reported so far and minimal literature described the role of these molecules. The presence of a glucose-2-phosphate has recently been reported in glycogen using NMR [33]. Phosphate incorporation (glucose C2 and C3 phosphomonoesters) into glycogen results from a catalytic error of the glycogen synthase leading to insoluble glycogen-like polymer in the Lafora disease. Another important biological role of the pyranose-2-phosphate motif concerns the quorum-sensing signal synthesis and dissemination of the Ti plasmid in agrobacteria which are under the control of the master transcriptional regulator AccR [12]. Once imported by AccA-ABC transporter into agrobacteria, agrocinopine A is degraded by the enzyme AccF into L-arabinose-2-phosphate and sucrose [15]. A single study based on gel-shift assays with AccR has proposed a direct binding between agrocinopine A and AccR [14] using a mixture of agrocinopines A and B purified from tomato tumours under acidic conditions (pH 5.4). We hypothesize that the gel shift result can be accounted for by the presence of L-arabinose-2-phosphate in the mixture. Here, we worked with synthetic agrocinopine A, L-arabinose-2-phosphate and D-glucose-2-phosphate. We show that (1) AccR interacts with L-arabinose-2-phosphate and D-glucose-2-phosphate and not with agrocinopine A; (2) agrocinopine A, L-arabinose-2-phosphate and D-glucose-2-phosphate induce the quorum-sensing signal synthesis and Ti plasmid transfer in wild-type *A*. *tumefaciens*; (3) the quorum-sensing signal synthesis and Ti plasmid transfer are abolished in an *accF* mutant of pTiC58 in the presence of agrocinopine A; but are still activated in the presence of L-arabinose-2-phosphate. Our work demonstrates the key-role of AccF in the maturation of L-arabinose-2-phosphate which triggers the quorum-sensing signal and *acc* operon expression. In the absence of L-arabinose-2-phosphate, the operon *acc* is not over-expressed leading to a weak expression of AccA thus to a weak import of agrocinopine. This can explain why the *accF* mutant failed to remove detectable amounts of a mixture of agrocinopines A and B from the media [17]. However, the same publication also showed that the *accF* mutant still took up agrocin 84. Therefore, from these results and from our data, AccF is not directly required for agrocinopine A transport. Our work refreshes the knowledge on the regulatory cascade of the quorum-sensing activation by the opine agrocinopine A in *A*. *tumefaciens*. It is important to keep in mind that AccF is surely involved in the maturation of agrocin 84 into the toxic moiety TM84 and D-glucose-2-phosphate. Altogether, our work supports the hypothesis that agrocin 84 hijacks the transport process of agrocinopine A *via* AccA and that D-glucose-2-phosphate has the same role than L-arabinose-2-phosphate in the quorum-sensing pathway of *A*. *tumefaciens* C58 pathogen and likely in the *acc* operon expression.

Agrocinopine A is the archetype of the agrocinopine family which encompasses the agrocinopines A, B, C and D [8]. Agrocinopine C is quite similar to agrocinopine A, as it is composed of a sucrose linked to a D-glucose (instead of a L-arabinose in agrocinopine A) via a phosphodiester bond. Agrocinopines B and D differ from A and C respectively, by lacking one sugar from the sucrose moiety. Agrocinopines A and B were often extracted and purified at acidic pH condition from tumours induced by *A*. *tumefaciens* strains such as C58 while the agrocinopines C and D from tumours induced by *A*. *tumefaciens* strains such as Bo542. One study has shown that radioactive agrocinopine A was detected from C58 tumours without the presence of agrocinopine B indicating that agrocinopine A is probably the sole agrocinopine secreted in tumours [16]. Moreover, agrocinopines B and D seem to be degradation products of agrocinopines A and C respectively, especially considering the latter two opines can be easily degraded either chemically by mild acid hydrolysis or enzymatically by α-glucosidase [8]. It is likely that crown gall tumours synthesize and secrete only one agrocinopine either A or C depending on its Ti-plasmid type C58 or Bo542. The AccF by-product of agrocinopines C and D is D-glucose-2-phosphate as for agrocin 84. In *A*. *tumefaciens* C58 and Bo542, AccA and AccR proteins are highly conserved with 75% and 83% sequence identity respectively, suggesting that they present the same function with a similar action mechanism. Therefore, L-arabinose-2-phosphate and D-glucose-2-phosphate must share a similar crucial role in all the agrocinopine pTi strains. In *A*. *tumefaciens* strain C58, we show that quorum-sensing and plasmid Ti transfer is activated by either L-arabinose-2-phosphate or D-glucose-2-phosphate. Moreover, a similar affinity of these compounds for AccR suggests that, although synthesis of agrocinopines A and C are induced by different T-DNAs (from pTiC58 and pTiTiBo542 respectively), they play a similar role in *A*. *tumefaciens* C58. Our work provides evidence of the functional redundancy among opines of the agrocinopine family. *A*. *radiobacter* K84 employs a smart strategy when it produces agrocin 84 taking advantage of the functional and structural similarity with the opines utilized by the agrobacteria harboring the agrocinopine-family Ti plasmids. The D-glucose-2-phosphate moiety of agrocin 84 acts as a ‘molecular passkey’, which is recognized by a large variety of pathogenic agrobacteria expressing the PBP AccA and enzyme AccF. This feature may explain the success story of *A*. *radiobacter* K84 as a biocontrol agent against *A*. *tumefaciens* pathogens [5,6].

In conclusion, the pyranose-2-phosphate found in the L-arabinopyranose moiety in agrocinopine A (and other tested analogues and derivatives) and the D-glucopyranose moiety in agrocin 84 and agrocinopine C, is a specific motif that possesses at least two functions (transport and regulation) in agrobacteria. In addition to being the mature signal that triggers quorum-sensing and further dissemination of virulence genes, it is the key-recognition motif of the PBP AccA presumably allowing the importation of toxic or non-toxic molecules as long as they possess it at one end.

## Materials and Methods

### Synthesis of agrocinopine A, agrocinopine 3’-*O*-benzoate, L-arabinose-2-phosphate and L-arabinose-2-isopropylphosphate

Agrocinopine A (**9**) was prepared according to an adapted synthesis which was previously reported by Lindberg and Norberg [34]. The synthesis sequence was modified by using a different phosphoramidite for the coupling step, in order to keep the phosphate group protected as an ester until the latest stage of the synthesis, easing the purification of the intermediate products by simple silica gel chromatography. Benzyl 3,4-*O*-isopropylidene-β-L-arabinoside **1** prepared in two steps from L-arabinose [35] was phosphinylated using phosphoramidate **2** [36,37] giving the 2-substituted arabinose derivative **3** in 79% yield, which was coupled in the presence of tetrazole to the partially protected sucrose derivative **4** having only its 4-OH unprotected prepared from sucrose in 4 steps [38]. The coupling product **5** obtained in 64% yield was oxidized with *t*-butylhydroperoxide forming the fully protected agrocinopine **6** in 92% yield. Deprotection of the three isopropylidene groups in 60% aq. AcOH at 50°C afforded compound **7** in 53% yield. Compound **7** was further debenzylated at the anomeric position of the arabinose and on the phosphate group by catalytic hydrogenation (Pd/C, H2) leading in 83% to compound **8** having only esters as remaining protecting groups (two acetyl groups at position 3 of the glucose moiety and position 6 of the fructose moiety, and one benzoyl group at position 3 of the fructose moiety). Final ester groups cleavage was performed using potassium carbonate in MeOH leading to agrocinopine (**9**) in 59% yield, for which all spectroscopic data were consistent with reported ones [39]. When sodium methoxide was used and the reaction stopped before total consumption of intermediate products, 3’-*O*-benzoylated agrocinopine (**10)** could be obtained as a mixture with agrocinopine. The L-arabinose derivatives **14** and **18** substituted at position 2 with a phosphate or an isopropylphosphate, respectively, were prepared from the same starting phosphinylated L-arabinose **3** following a similar sequence. Reaction of **3** with benzyl alcohol or isopropanol followed by oxidation, hydrolysis of the isopropylidene group and catalytic hydrogenation of the benzyl groups led to the desired L-arabinose derivatives **14** and **18** respectively (Fig 2). Details on the procedures and spectroscopic data are given in S1 Text.

### Synthesis of d-glucose-2-phosphate

The synthesis of glucose-2-phosphate was based on a strategy involving the selective deprotection in position 2 of perbenzylated glucose and then phosphorylation. This synthesis was achieved starting from D-glucose in five steps. First, the benzyl glucoside **19** was obtained using sulfamic acid in benzyl alcohol to get the α-isomer as the major one [40]. Compound **19** was then fully benzylated to obtain compound **20** which was selectively deprotected in position 2 using TIBAL. This method described by Sinaÿ *et al*. can only debenzylate the α-isomer of 20 and led to compound **21** [41] which was then phosphorylated using the dibenzyl phosphoramidite and subsequently oxidized to compound **22**. Glucose-2-phosphate was finally obtained by deprotection of benzyl groups using palladium catalyzed hydrogenation, as a mixture of α/β isomers, the α isomer being the major one. The structure of glucose-2-phosphate was ascertained by mass spectrometry and 2D-NMR experiments; in particular, the position 2 of the phosphate could be confirmed by 31P-1H correlations.

### Purification of agrocin 84

A 3 L starter culture of *A*. *tumefaciens* NT1 (pAgK84::Tn5 *A1-B5*) agrocin over producing strain was inoculated into 297 L of D-glucose minimal media (modified from Richaud *et al*.)[18] containing kanamycin at 50 μg/mL and incubated in a large scale fermenter at 28°C for 24 h, at a low aeration rate and moderate agitation (~250 rpm). The final culture was centrifuged at 9600 *g* and the supernatant containing agrocin 84 was retained. Activated Nuchar SN-20 charcoal (MeadeWestvaco Corporation) was added to the supernatant at 10 g per L and stirred at 4°C for 15 min. The agrocin 84 bound charcoal was allowed to settle, the supernatant removed and the charcoal slurry was vacuum filtered using a large Buchner Funnel and Whatman filter paper No. 3. 60 g portions of charcoal were then washed with 6 L of ddH2O at 4°C for 15 minutes and then re-filtered. Agrocin 84 was then eluted from the charcoal using 600 mL of 70% ethanol (per 60 g of charcoal) or reagent alcohol and stirring the solution overnight at 4°C. The agrocin 84 containing ethanol was vacuum, then filtered using Whatman paper No. 3 and then Millipore White Nylon 0.20 μm filters and the filtrate collected. The presence of agrocin 84 in samples was confirmed through use of an agrocin 84 bioassay [6]. Rotary evaporation was then used to remove ethanol and H2O and to concentrate the sample to a smaller volume. Methanol was added to each sample to a final concentration >70% and the resulting precipitate (containing insoluble contaminants) was removed by centrifugation of samples for 10 min at 17000 g and decanting the agrocin 84 containing supernatant. Remaining alcohol was removed by speed-vac or freeze dryer and samples stored at -80°C.Agrocin 84 was then purified using Reverse phase HPLC using a Clipeus C18 column (5 μm, 250 x 10 mm, Higgins Analytical, Inc) and the following typical conditions: buffer A (25 mM TEAA pH 7.5, in 100% H2O); buffer B (25 mM TEAA pH 7.5, in 50% H2O/ 50% acetonitrile; gradient 9–19% mobile phase buffer B over 33.5 min at a flow rate of 4.7 mL/min; and column temperature of 30°C. HPLC runs were monitored by UV absorbance at 264 nm and collected peaks showing activity in agrocin 84 bioassays were pooled. If necessary, samples were re-purified by HPLC, desalted, and the agrocin 84 concentration determined using agrocin 84 extinction coefficient [32]. Samples were stored at -80°C until use.

### Cloning, expression and purification of mature AccA

The mature AccA expression plasmid was obtained by cloning *accA* gene, without the 29 residues signal sequence, of *A*. *tumefaciens* C58 by PCR and adding a C-terminal hexahistidine tag into the plasmid pET-9aSN1 (a gift from S. Chéruel, I2BC, University Paris Sud, Orsay, France) between the *Nde*I and *Not*I sites using 5’GGATTCCATATGCAAGAACGCCGGGCGCTT3’ as forward primer and 5’TTTGCGGCCGCTCAATGGAGAGTGATGGTGATGGTGGCCGAAGCTGAGATTGTT3’ as reverse primer. *E*. *coli* BL21 competent cells transformed with pET9aSN1-AccA were grown in 2TY media at 37°C until OD600 of 0.6. 0.5 mM of isopropyl β-D-1-thiogalactopyranoside (IPTG) was added to the culture for 3 h of expression. The cells were pelleted by centrifugation at 8000 g for 20 min at 4°C and stored at -20°C. For protein purification, the cells are resuspended in 50 mM Tris-HCl pH 8, 150 mM NaCl and 20 mM imidazole and disrupted by sonication. After centrifugation at 25000 g for 45 minutes, the filtered supernatant is injected on a nickel affinity column (HiTrap 5 ml, GE Healthcare). After a washing step of 6% of 50 mM Tris-HCl pH 8, 150 mM NaCl and 300 mM imidazole (Buffer B), the protein is eluted with 100% of Buffer B and injected on a gel filtration Superdex 200 26/60 (GE Healthcare) using 50 mM Tris-HCl pH 8 and 150 mM NaCl. The protein fractions are pooled, concentrated and stored at -80°C.

### Expression and purification of mature seleniated AccA

The *E*. *coli* BL21 cell transformed with the plasmid pET9aSN1-AccA were grown overnight at 28°C in M9 media supplemented with 0.4% Glucose, 2 mM MgSO4, 1 μM CaCl2, 100 mg/L of lysine, threonine, and phenylalanine, 50 mg/L of leucine, valine, isoleucine and methionine. The pelleted cells are resuspended in fresh M9 media (same as above) with 100 mg/L of selenomethionine instead of methionine for 1 h at 37°C before inducing the expression with 0.5 mM IPTG for 5 hours. The cells are are centrifuged at 8000 g for 20 min at 4°C and stored at -20°C. The purification protocol was the same as described above.

### Expression and purification of AccR

The native AccR sequence was chemically synthesized (Genscript, Piscataway, NJ) with the addition of a 6-His tag at the C terminus and of two restriction sites *Nde*I and *Not*I at the N and C terminus respectively. The open reading frames were inserted into the pET-9aSN1 expression plasmid as for *accA* ORF.

*E*. *coli* C41 competent cells transformed with pET9aSN1-AccR were grown in LB media at 37°C until OD600 of 0.6. 1 mM of IPTG was added to the culture for 20 h of expression at 20°C. The cells were pelleted by centrifugation at 8000 g for 20 min at 4°C and stored at -20°C. For protein purification, the cells were resuspended in 20 mM Bis Tris propane pH 9, 150 mM NaCl, 10% glycerol (Buffer C) and disrupted by sonication. After centrifugation at 25000 g for 45 minutes, the filtered supernatant was injected on a nickel affinity column (cOmplete His-Tag Purification Column, 5ml, Roche Lif Sciences). After a washing step using buffer C with 5 mM imidazole, the protein was eluted with 100% of buffer C with 100 mM imidazole before a dialysis against buffer C. The protein was then concentrated and stored at -80°C.

### Crystallization and data collection of AccA

Crystallization conditions for AccA at 12 mg/mL in presence and absence of ligands (between 50 μM and 10 mM) were screened using Qiagen kits (Valencia, CA, USA) with a Cartesian nanodrop robot (Genomic solutions). The crystals were manually reproduced in hanging drops experiments by mixing equal volumes of protein solution and precipitant solution mentioned in Table 1. Crystals were transferred to a cryoprotectant solution (paraffin oil or mother liquor supplemented with 25% PEG 400) and flash-frozen in liquid nitrogen. X-ray diffraction data sets were collected at 100 K on the Proxima 1 beamline (SOLEIL synchrotron, Saint-Aubin, France). Data processing was performed using the XDS package [42] (Table 1).

### Structure determination and refinement of AccA

The crystal structure of the AccA-Agrocinopine complex was determined by SAD method from selenomethionine-labelled protein and refined at 2.65 Å resolution. Solvent content analysis using CCP4 (Collaborative Computational Project, Number 4) indicated the presence of one monomer in the asymmetric unit. The positions of 17 over 18 selenium atoms were found using SHELX suite program [43]. The phases were calculated using PHASER [44] and density modification was performed by PARROT (CCP4 suite). An iterative process of manual building in COOT combined with phase calculation using PHASER [44], where a partial model was used as input, allowed the modelling of the complete polypeptide chain. The structure of the free-liganded AccA was solved by molecular replacement with PHASER [44] using the coordinates of lobe 1 and lobe 2 of SeMet-AccA monomer as separated search models whereas all the complexed structures were solved using the SeMet-AccA monomer as a search model. Refinement of each structure was performed with BUSTER-2.10 [45] with NCS restraints when the asymmetric unit contains more than one protein molecule. One TLS group was assigned for each structure. Inspection of the density maps and manual rebuilding were performed using COOT [46]. The three dimensional models of agrocinopine, agrocin 84 and agrocinopine 3’-*O*-benzoate, L-arabinose-2-phosphate and L-arabinose-2-isopropylphosphate were generated using the ProDRG webserver [47]. Refinement details of each structure are shown in Table 1. Molecular graphics images were generated using PyMOL (http://www.pymol.org).

### Fluorescence titration measurements of AccA

Each ligand bound to AccA was monitored by autofluorescence by excitating the protein at a wavelength of 295 nm and monitoring the quenching of fluorescence emission of tryptophans at 335 nm. All experiments were performed at 22°C in 3 x 15 mm quartz cuvettes using Cary Eclypse spectrofluorometer (Varian) in 25 mM Tris-HCl pH 8.0 and 150 mM NaCl with a fixed amount of proteins (2 μM) and increasing concentrations of ligand. Each ligand has no emission signal at 335 nm. The data were analysed using Origin 7 software and fitted to the equation f = ΔFluorescencemax*abs(x)/(*K*_*D*_+abs(x)).

### Isothermal titration microcalorimetry measurements of AccA and AccR

Isothermal titration microcalorimetry experiments were performed with an ITC200 isothermal titration calorimeter from MicroCal (GE Healthcare). The experiments were carried out at 20°C for AccA and 25°C for AccR. Protein concentration in the microcalorimeter cell (0.2 mL) varied from 10 to 25 μM for AccA and 40 μM for AccR. 19 injections of 2 μl of ligand solution (agrocinopine A, agrocinopine 3’-*O*-benzoate, L-arabinose-2-isopropylphosphate and L-arabinose-2-phosphate) concentration from 150 to 240 μM for AccR and from 300 to 500 μM for AccR were performed at intervals of 180 s while stirring at 500 rpm. For agrocin 84 experiment, we inverted the protein and ligand positions: the ligand was in the cell and the protein in the syringe. This strategy was chosen to overcome the small amount of agrocin 84. The experimental data were fitted to theoretical titration curves with software supplied by MicroCal (ORIGIN). This software uses the relationship between the heat generated by each injection and ΔH (enthalpy change in Kcal.Mol-1), Ka (the association binding constant in M-1), n (the number of binding sites), total protein concentration and free and total ligand concentrations [48].

### Agrocinopine 3’-*O*-benzoate uptake assay

A single colony of *Agrobacterium tumefaciens* C58 with pTiC58 derivative pTi::Gm [49] was grown overnight at 28°C in AB media supplemented with mannitol (2 g/L) and gentamicin 25 μg.ml-1. 20 ml of AB media in presence of a carbon source (agrocinopine 3’-*O*-benzoate or L-arabinose-2-phosphate at a final concentration of 1 mM) or in absence of any carbon source and supplemented with gentamicin (25 μg.ml-1) were inoculated at an initial OD600 of 0.03 and the OD was monitored for 48 hours.

### *A*. *tumefaciens* strains and growth conditions

The *A*. *tumefaciens* C58 control is the pTiC58 derivative pTi::Gm [49] and the defective *accF* mutant (*A*. *tumefaciens* C58 accF) is a kind gift from S. Farrand [14]. In complementation assay, the *accF* wild-type gene was cloned into p6000 [50] plasmid and electroporated into the *accF* mutant. Rifampicin-resistant recipient strain C58.00 was derived from *A*. *tumefaciens* C58 cured of the At and Ti plasmids[51]. *A*. *tumefaciens* was cultivated at 28°C in AB minimal medium containing ammonium chloride (1 g/L) and mannitol (2 g/L), or in Luria-Bertani modified medium (LBm; NaCl 5 g/L). The antibiotics gentamycin and rifampicin were added at 25 μg/mL and 100 μg/mL, respectively.

### Conjugation assays and quantification of OC8HSL

Overnight LBm cultures of recipient (C58.00) and donor (C58 control) cells were mixed in an equal ratio (1:1). 10 μl of this mix were transferred into 140 μl of AB medium supplemented with mock or 1 mM of agrocinopine A, L-arabinose-2-phosphate and glucose-2-P for static liquid cultures of 24, 48, 72 and 96 hours. Suspension dilutions of these cultures were spotted onto selective agar media to enumerate the different bacterial populations (donor, recipient and Ti plasmid transconjugants). In parallel, to quantify OC8HSLs, aliquots of cell cultures were spotted onto TLC plates (RP-18/UV254, Macherey-Nagel) and incubated with the OC8HSL-bioindicator strain *A*. *tumefaciens* NT1(pZLR4) as previously described [52]. Tested samples were compared with a calibration curve obtained with pure OC8HSL (Sigma-Aldrich).

### Accession numbers

Coordinates and structure factors have been deposited at the Protein Data Bank (PDB) under accession codes 4ZE9 (seleniated AccA with agrocinopine), 4ZE8 (free-liganded AccA), 4ZEB (AccA with agrocinopine), 4ZEC (AccA with agrocin 84), 4ZED (AccA with agrocinopine 3’-*O*-benzoate), 4ZEK (AccA with L-arabinose-2-isopropylphosphate), 4ZEI (AccA with L-arabinose-2-phosphate) and 4RA1 (AccA with D-glucose-2-phosphate)

## Supporting Information

S1 Fig Ligand bound to the ligand binding site of AccA in their annealing Fo-Fc omit map contoured at 4 σ (a) agrocinopine A, (b) agrocin 84, (c) agrocinopine 3’-*O*-benzoate, (d) L-arabinose-2-isopropylphosphate, (e) L-arabinose-2-phosphate, (f) D-glucose-2-phosphate. (PDF)

S2 Fig **OH1 bound in AccA binding site a**. Stick representation of AccA residues interacting with arabinose O1H group in agrocinopine A (in yellow). Residues from the lobe 1, lobe 2 and hinge region are shown in slate, pink and red, respectively. **b**. Superimposition of three bound ligands, agrocinopine A (yellow), agrocin 84 (orange), agrocinopine 3’-*O*-benzoate (purple) are shown as stick.
(PDF)

S3 Fig Agrocinopine benzoate is used as a carbon source. OD monitoring (600 nm) of cultures in presence of agrocinopine 3’-*O*-benzoate (black squares) L-arabinose-2-phosphate (black circles) as a carbon source and in absence of any carbon source (black triangles) in AB minimum media.
(PDF)

S4 Fig AccA fluorescence monitoring upon titration with each ligand and fit (solid line) to a single binding model using Origin. Measures were done in triplicates.
(PDF)

S5 Fig AccA microcalorimetry measurements. The top panels show heat differences upon injection of ligand and lower panels show integrated heats of injection and the best fit (solid line) to a single binding model using Microcal Origin.
(PDF)

S6 Fig Structural comparison of the ligand binding sites. **(a)**. between AccA (grey)-agrocinopine A and DPP (green)-GL complexes. Agrocinopine A and GL are shown in cyan and orange, respectively. The loop region 402–414 in AccA shown in green corresponds to the helix 383–399 from DPP shown in grey. **(b)** between AccA (grey)-agrocinopine A and DPP (green)-GL complexes. Agrocinopine A and GL are shown in cyan and orange, respectively. The two tryptophans gate (Trp178 and Trp423) and the loop 372–378 from AccA are represented in red while the corresponding residues (Met152 and Asp408) and the loop 351–359 in DPP are in deep blue.
(PDF)

S7 Fig Ligand binding sites of tmCBP (a) and AccA (b). tmCBP (PDB ID 2O7I & 4JSO) and AccA are colored in green and slate respectively. Agrocinopine A (yellow), cellobiose (light blue) and laminaripentaose (green) are represented as sticks.
(PDF)

S1 Table Autofluorescence affinity results. The *K*_*D*_ values were obtained using Microcal Origin. and fitting to a one binding site model using the following equation: f = ΔFluorescencemax*abs(x)/(*K*_*D*_+abs(x)). ND: no signal detected.
(PDF)

S2 Table Microcalorimetry results. The values were obtained using Microcal Origin. and fitting to a one binding site model. No signal detected for L-arabinose and D-glucose.
(PDF)

S1 Text Detailed description of molecules synthesis. Chemical synthesis and characterisation of synthetized molecules are described.
(PDF)

## References

[1] Berntsson RP, Smits SHJ, Schmitt L, Slotboom D-J, Poolman B. A structural classification of substrate-binding proteins. FEBS Lett. 2010; 584: 2606–17. 10.1016/j.febslet.2010.04.043 20412802

[2] Roberts WP, Tate ME, Kerr A. Agrocin 84 is a 6-N-phosphoramidate of an adenine nucleotide analogue. Nature. 1977; 265: 379–81. 834287 10.1038/265379a0

[3] Thompson RJ, Hamilton RH. H, Pootjes C. FF. Purification and characterization of agrocin 84. Antimicrob Agents Chemother. 1979; 16: 293–6. 507786 10.1128/aac.16.3.293 PMC352848

[4] Gelvin SB. Agrobacterium-mediated plant transformation: the biology behind the “gene-jockeying” tool. Microbiol Mol Biol Rev. 2003; 67: 16–37. 12626681 10.1128/MMBR.67.1.16-37.2003 PMC150518

[5] Kim J-G, Park BK, Kim S-U, Choi D, Nahm BH, Moon JS, et al Bases of biocontrol: sequence predicts synthesis and mode of action of agrocin 84, the Trojan horse antibiotic that controls crown gall. Proc Natl Acad Sci U S A. 2006; 103: 8846–51. 16731618 10.1073/pnas.0602965103 PMC1482666

[6] Reader JS, Ordoukhanian PT, Kim J, Hwang I, Farrand S. Major Biocontrol of Plant Tumors. Science (80-). 2005; 309: 1533. 10.1126/science.1116841 16141066

[7] Chopra S, Palencia A, Virus C, Tripathy A, Temple BR, Velazquez-Campoy A, et al Plant tumour biocontrol agent employs a tRNA-dependent mechanism to inhibit leucyl-tRNA synthetase. Nat Commun. 2013; 4: 1417 10.1038/ncomms2421 23361008

[8] Ellis JG, Murphy PJ. Four new opines from crown gall tumours—Their detection and properties. Mol Gen Genet. 1981; 181: 36–43.

[9] Veselov D, Langhans M, Hartung W, Aloni R, Feussner I, Gotz C, et al Development of *Agrobacterium tumefaciens* C58-induced plant tumors and impact on host shoots are controlled by a cascade of jasmonic acid, auxin, cytokinin, ethylene and abscisic acid. Planta. 2003; 216: 512–522. 12520344 10.1007/s00425-002-0883-5

[10] Chilton MD, Saiki RK, Yadav N, Gordon MP, Quetier F. T-DNA from *Agrobacterium* Ti plasmid is in the nuclear DNA fraction of crown gall tumor cells. Proc Natl Acad Sci U S A. 1980; 77: 4060–4. 16592850 10.1073/pnas.77.7.4060 PMC349769

[11] Hernalsteens JP, Thia-Toong L, Schell J, Van Montagu M. An *Agrobacterium*-transformed cell culture from the monocot *Asparagus officinalis* . EMBO J. 1984; 3: 3039–41. 16453585 10.1002/j.1460-2075.1984.tb02254.x PMC557813

[12] Beck von Bodman S, Hayman GT, Farrand SK. Opine catabolism and conjugal transfer of the nopaline Ti plasmid pTiC58 are coordinately regulated by a single repressor. Proc Natl Acad Sci U S A. 1992; 89: 643–7. 1731335 10.1073/pnas.89.2.643 PMC48295

[13] Ellis JG, Kerr A, Petit A, Tempe J. Conjugal transfer of nopaline and agropine Ti-plasmids ? The role of agrocinopines. Mol Gen Genet. 1982; 186: 269–274.

[14] Kim HS, Yi H, Myung J, Piper KR, Farrand SK. Opine-based *Agrobacterium* competitiveness: dual expression control of the agrocinopine catabolism (acc) operon by agrocinopines and phosphate levels. J Bacteriol. 2008; 190: 3700–11. 10.1128/JB.00067-08 18344359 PMC2395003

[15] Kim H, Farrand SK. Characterization of the acc operon from the nopaline-type Ti plasmid pTiC58, which encodes utilization of agrocinopines A and B and susceptibility to agrocin 84. J Bacteriol. 1997; 179: 7559–72. 9393724 10.1128/jb.179.23.7559-7572.1997 PMC179710

[16] Messens E, Lenaerts A, Hedges RW, Montagu M V. Agrocinopine A, a phophorylated opine is secreted from crown gall cells. EMBO J. 1985; 4: 571–577. 15926217 10.1002/j.1460-2075.1985.tb03668.x PMC554227

[17] Ryders MH, Tate ME, Jones P, Tateq ME, Jonesn P. Agrocinopine A, a tumor inducing Plasmid-coded enzyme product, is a Phosphodiester of Sucrose and L-arabinose. J Biol Chem. 1984; 259: 9704–9710. 6746666

[18] Richaud C, Mengin-Lecreulx D, Pochet S, Johnson E, Cohen G, Marliere P. Directed evolution of biosynthetic pathways. Recruitment of cysteine thioethers for constructing the cell wall of *Escherichia coli* . J Biol Chem. 1993; 268: 26827–26835. 8262915

[19] Tate ME, Murphy PJ, Roberts WP, Keer A. Adenine N6-substituent of agrocin 84 determines its bacteriocin-like specificity. Nature. 1979; 280: 697–9. 471050 10.1038/280697a0

[20] Kerr A, Tate ME. Agrocin 84 Mal Otis C. Encyclopedia of Plant Pathology. 2001 pp. 20–21.

[21] Tate M. E, Kerr A. Encyclopedia of Agrochemicals. Plimmer JR, Gammon DW, Ragsdale NA, editors. 2003.

[22] Krissinel E, Henrick K. Secondary-structure matching (SSM), a new tool for fast protein structure alignment in three dimensions. Acta Crystallogr D Biol Crystallogr. 2004/12/02 ed. 2004; 60: 2256–68. 15572779 10.1107/S0907444904026460

[23] Lebrette H, Borezée-Durant E, Martin L, Richaud P, Boeri Erba E, Cavazza C. Novel insights into nickel import in *Staphylococcus aureus*: the positive role of free histidine and structural characterization of a new thiazolidine-type nickel chelator. Metallomics. 2015; 10.1039/c4mt00295d 25611161

[24] Levdikov VM, Blagova E V, Brannigan J a, Wright L, Vagin A a, Wilkinson AJ. The structure of the oligopeptide-binding protein, AppA, from *Bacillus subtilis* in complex with a nonapeptide. J Mol Biol. 2005; 345: 879–92. 15588833 10.1016/j.jmb.2004.10.089

[25] Tame JR, Sleigh SH, Wilkinson AJ, Ladbury JE. The role of water in sequence-independent ligand binding by an oligopeptide transporter protein. Nat Struct Biol. 1996; 3: 998–1001. 8946852 10.1038/nsb1296-998

[26] Klepsch MM, Kovermann M, Löw C, Balbach J, Permentier HP, Fusetti F, et al *Escherichia coli* peptide binding protein OppA has a preference for positively charged peptides. J Mol Biol. 2011; 414: 75–85. 10.1016/j.jmb.2011.09.043 21983341

[27] Sleigh SH, Seavers PR, Wilkinson AJ, Ladbury JE, Tame JR. Crystallographic and calorimetric analysis of peptide binding to OppA protein. J Mol Biol. 1999; 291: 393–415. 10438628 10.1006/jmbi.1999.2929

[28] Lebrette H, Iannello M, Fontecilla-Camps JC, Cavazza C. The binding mode of Ni-(L-His)2 in NikA revealed by X-ray crystallography. J Inorg Biochem. 2013; 121: 16–8. 10.1016/j.jinorgbio.2012.12.010 23314594

[29] Cuneo MJ, Beese LS, Hellinga HW. Structural analysis of semi-specific oligosaccharide recognition by a cellulose-binding protein of *Thermotoga maritima* reveals adaptations for functional diversification of the oligopeptide periplasmic binding protein fold. J Biol Chem. 2009; 284: 33217–23. 10.1074/jbc.M109.041624 19801540 PMC2785164

[30] Dunten P, Mowbray SL. Crystal structure of the dipeptide binding protein from *Escherichia coli* involved in active transport and chemotaxis. Protein Sci. 1995; 4: 2327–34. 8563629 10.1002/pro.5560041110 PMC2143009

[31] Hayman GT, Beck von Bodman S, Kim H, Jiang P, Farrand SK. Genetic analysis of the agrocinopine catabolic region of *Agrobacterium tumefaciens* Ti plasmid pTiC58, which encodes genes required for opine and agrocin 84 transport. J Bacteriol. 1993; 175: 5575–84. 8366042 10.1128/jb.175.17.5575-5584.1993 PMC206614

[32] Murphy PJ, Tate ME, Kerr A. Substituents at N6 and C-5′ Control Selective Uptake and Toxicity of the Adenine-Nucleotide Bacteriocin, Agrocin 84, in Agrobacteria. Eur J Biochem. 2005; 115: 539–543. 10.1111/j.1432-1033.1981.tb06236.x 6263633

[33] Tagliabracci VS, Heiss C, Karthik C, Contreras CJ, Glushka J, Ishihara M, et al Phosphate incorporation during glycogen synthesis and Lafora disease. Cell Metab. 2011; 13: 274–82. 10.1016/j.cmet.2011.01.017 21356517 PMC3124772

[34] Lindberg M, Norberg T. Synthesis of Sucrose 4′-(L-Arabinose-2-YL Phosphate) (Agrocinopihe A) Using an Arabinose 2-H-Phosphonate Intermediate. J Carbohydr Chem. 1988; 7: 749–755.

[35] Rölle T, Hoffmann RW. Model Studies towards the Synthesis of the Right-Hand Part of Pederin. Helv Chim Acta. 2004; 87: 1214–1227.

[36] Bannwarth W, Trzeciak A. A Simple and Effective Chemical Phosphorylation Procedure for Biomolecules. Helv Chim Acta. 1987; 70: 175–186.

[37] Buijsman RC, Basten JEM, Dreef-Tromp CM, Marel GA, Boeckel CAA, Boom JH. Synthesis of heparin-like antithrombotics having perphosphorylated thrombin binding domains. Bioorg Med Chem. 1999; 7: 1881–1890. 10530936 10.1016/s0968-0896(99)00139-x

[38] Clode DM, Laurie WA, McHale D, Sheridan JB. Synthesis of 6,1′,3′-, 2,6,1′-, 1′,3′,6′-, and 2,1′,6′-tri-O-benzoylsucrose. Carbohydr Res. 1985; 139: 161–183.

[39] Messens E, Lenaerts A, Montagu M Van, De Bruyn A, Jans AWH, Binst G Van. P NMR Spectroscopy of Agrocinopine. J Carbohydr Chem. 1986; 5: 683–699.

[40] Guchhait G, Misra AK. Efficient glycosylation of unprotected sugars using sulfamic acid: A mild eco-friendly catalyst. Catal Commun. 2011; 14: 52–57.

[41] Lecourt T, Herault A, Pearce AJ, Sollogoub M, Sinaÿ P. Triisobutylaluminium and diisobutylaluminium hydride as molecular scalpels: the regioselective stripping of perbenzylated sugars and cyclodextrins. Chemistry. 2004; 10: 2960–71. 15214078 10.1002/chem.200305683

[42] Kabsch W. XDS. Acta Crystallogr D Biol Crystallogr. 2010; 66: 125–32. 10.1107/S0907444909047337 20124692 PMC2815665

[43] Sheldrick GM. A short history of SHELX. Acta Crystallogr A. 2008; 64: 112–22. 18156677 10.1107/S0108767307043930

[44] McCoy AJ, Grosse-Kunstleve RW, Adams PD, Winn MD, Storoni LC, Read RJ. Phaser crystallographic software. J Appl Crystallogr. 2007/8/01 ed. 2007; 40: 658–674. 19461840 10.1107/S0021889807021206 PMC2483472

[45] Blanc E, Roversi P, Vonrhein C, Flensburg C, Lea SM, Bricogne G. Refinement of severely incomplete structures with maximum likelihood in BUSTER-TNT. Acta Crystallogr D Biol Crystallogr. 2004; 60: 2210–21. 15572774 10.1107/S0907444904016427

[46] Emsley P, Cowtan K. Coot: model-building tools for molecular graphics. Acta Crystallogr D Biol Crystallogr. 2004/12/02 ed. 2004; 60: 2126–2132. 15572765 10.1107/S0907444904019158

[47] Schüttelkopf AW, Aalten DMF, van Aalten DMF. PRODRG: a tool for high-throughput crystallography of protein-ligand complexes. Acta Crystallogr D Biol Crystallogr. 2004; 60: 1355–63. 15272157 10.1107/S0907444904011679

[48] Wiseman T, Williston S, Brandts JF, Lin LN. Rapid measurement of binding constants and heats of binding using a new titration calorimeter. Anal Biochem. 1989/5/15 ed. 1989; 179: 131–137. 2757186 10.1016/0003-2697(89)90213-3

[49] Haudecoeur E, Planamente S, Cirou a, Tannières M, Shelp BJ, Moréra S, et al Proline antagonizes GABA-induced quenching of quorum sensing in *Agrobacterium tumefaciens* . Proc Natl Acad Sci U S A. 2009; 106: 14587–92. 10.1073/pnas.0808005106 19706545 PMC2732848

[50] Maurhofer M, Reimmann C, Schmidli-Sacherer P, Heeb S, Haas D, Défago G. Salicylic Acid Biosynthetic Genes Expressed in *Pseudomonas* fluorescens Strain P3 Improve the Induction of Systemic Resistance in Tobacco Against Tobacco Necrosis Virus. Phytopathology. 1998; 88: 678–684. 10.1094/PHYTO.1998.88.7.678 18944940

[51] Lang J, Planamente S, Mondy S, Dessaux Y, Moréra S, Faure D. Concerted transfer of the virulence Ti plasmid and companion At plasmid in the *Agrobacterium tumefaciens*-induced plant tumour. Mol Microbiol. 2013; 90: 1178–89. 10.1111/mmi.12423 24118167

[52] Cha C, Gao P, Chen YC, Shaw PD, Farrand SK. Production of acyl-homoserine lactone quorum-sensing signals by gram-negative plant-associated bacteria. Mol Plant Microbe Interact. 1998; 11: 1119–29. 9805399 10.1094/MPMI.1998.11.11.1119

