# Supplementary material for: A Pyranose-2-Phosphate Motif Is Responsible for Both Antibiotic Import and Quorum-Sensing Regulation in Agrobacterium tumefaciens
Source: PLoS Pathog. 2015 Aug 5;11(8):e1005071. doi: 10.1371/journal.ppat.1005071 (PMC4526662; doi:10.1371/journal.ppat.1005071)
Supplement: S1 Fig — (PDF) [file ppat.1005071.s001.pdf]

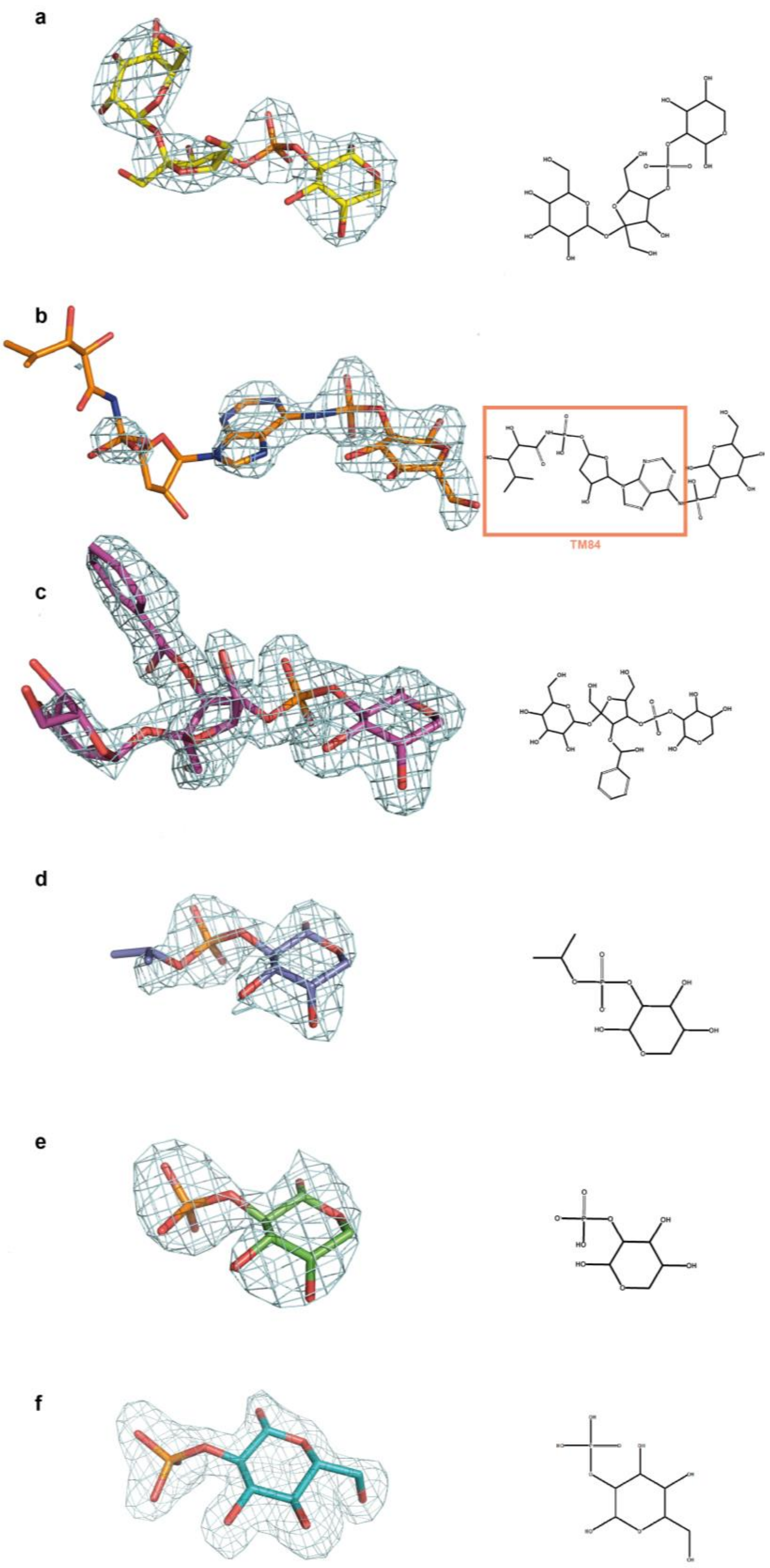

S1 Fig Ligand bound to the ligand binding site of AccA in their annealing Fo-Fc omit map contoured at 4 σ (a) agrocinopine A, (b) agrocin 84, (c) agrocinopine 3’-*O*-benzoate, (d) L-arabinose-2-isopropylphosphate, (e) L-arabinose-2-phosphate, (f) D-glucose-2-phosphate.
