# Supplementary material for: A Pyranose-2-Phosphate Motif Is Responsible for Both Antibiotic Import and Quorum-Sensing Regulation in Agrobacterium tumefaciens
Source: PLoS Pathog. 2015 Aug 5;11(8):e1005071. doi: 10.1371/journal.ppat.1005071 (PMC4526662; doi:10.1371/journal.ppat.1005071)
Supplement: S2 Fig — OH1 bound in AccA binding site a. Stick representation of AccA residues interacting with arabinose O1H group in agrocinopine A (in yellow). Residues from the lobe 1, lobe 2 and hinge region are shown in slate, pink and red, respectively. b. Superimposition of three bound ligands, agrocinopine A (yellow), agrocin 84 (orange), agrocinopine 3’-O-benzoate (purple) are shown as stick. (PDF) [file ppat.1005071.s002.pdf]

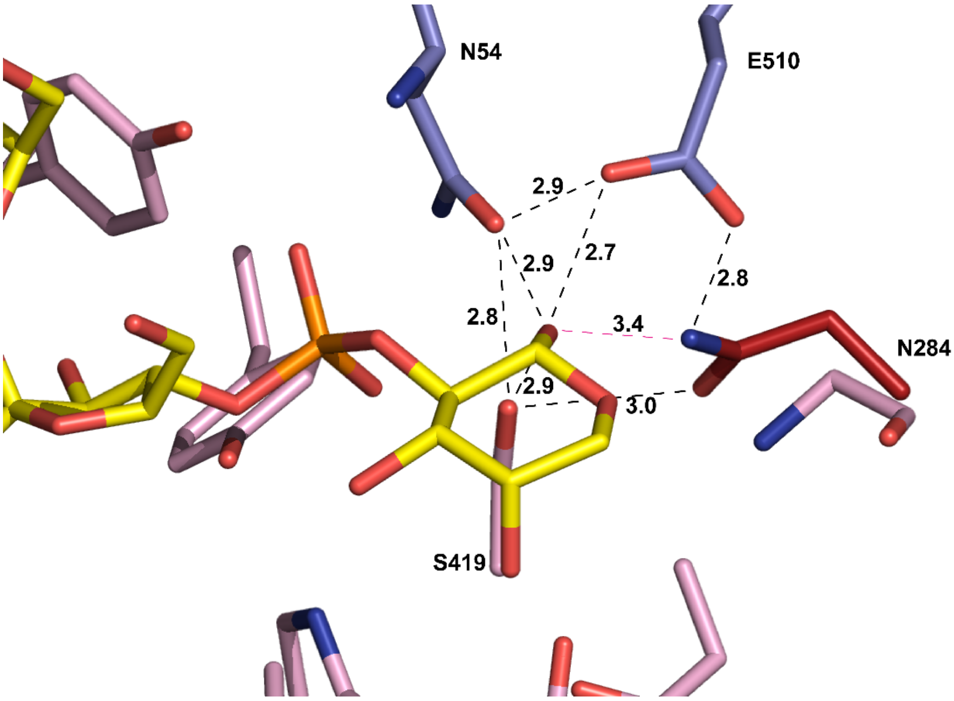

S2a Fig Stick representation of AccA residues interacting with arabinose O1H group in agrocinopine A (in yellow). Residues from the lobe 1, lobe 2 and hinge region are shown in slate, pink and red, respectively.

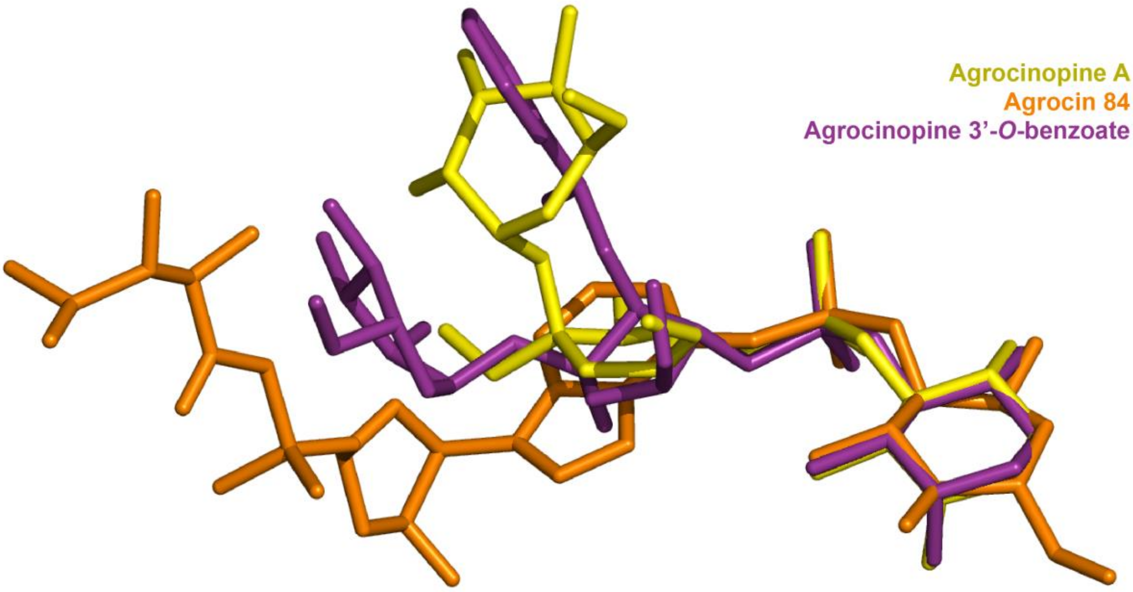

S2b Fig Superimposition of three bound ligands: agrocinopine A (yellow), agrocin 84 (orange), agrocinopine 3'-*O*-benzoate (purple) are shown as stick.
