# Supplementary material for: A Pyranose-2-Phosphate Motif Is Responsible for Both Antibiotic Import and Quorum-Sensing Regulation in Agrobacterium tumefaciens
Source: PLoS Pathog. 2015 Aug 5;11(8):e1005071. doi: 10.1371/journal.ppat.1005071 (PMC4526662; doi:10.1371/journal.ppat.1005071)
Supplement: S3 Fig — OD monitoring (600 nm) of cultures in presence of agrocinopine 3’-O-benzoate (black squares) L-arabinose-2-phosphate (black circles) as a carbon source and in absence of any carbon source (black triangles) in AB minimum media. (PDF) [file ppat.1005071.s003.pdf]

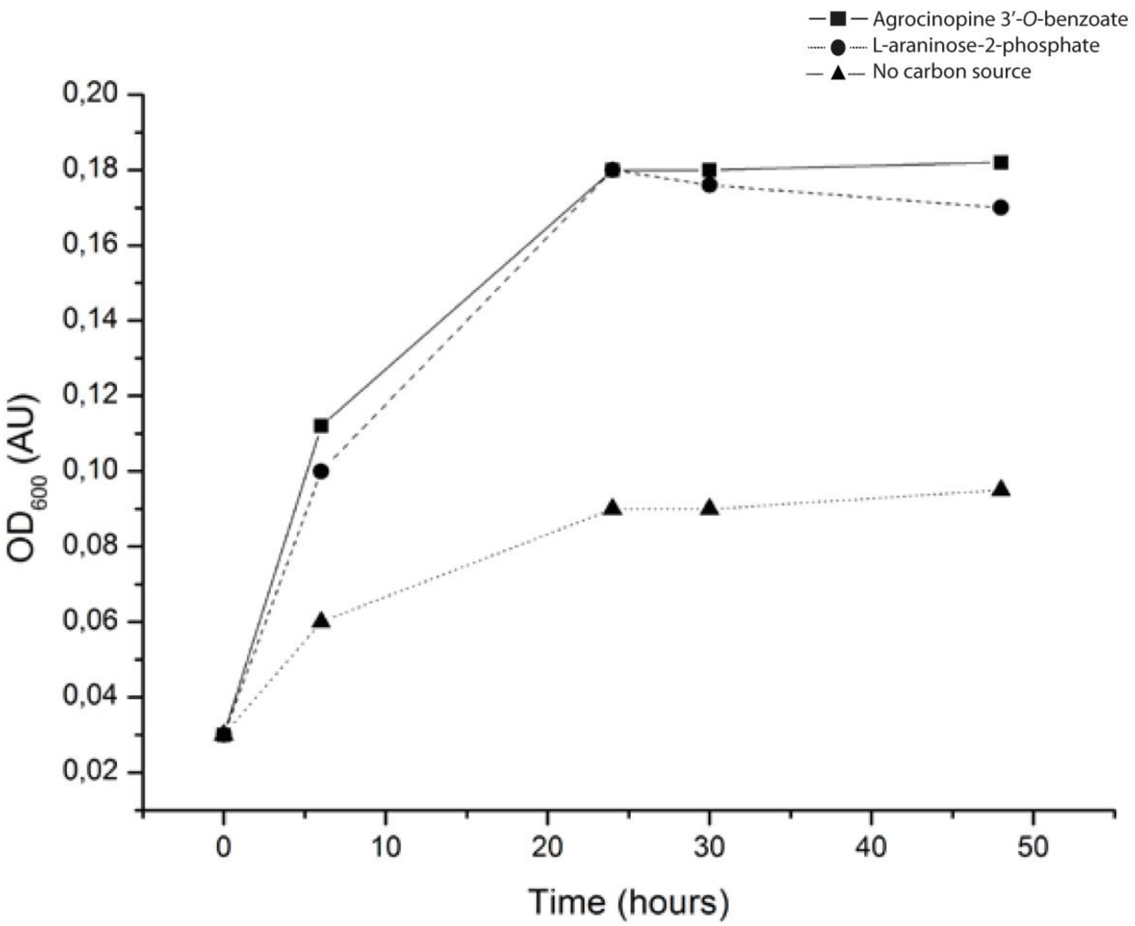

S3 Fig Agrocinopine benzoate is used as a carbon source. OD monitoring (600 nm) of cultures in presence of agrocinopine 3'-*O*-benzoate (black squares) L-arabinose-2-phosphate (black circles) as a carbon source and in absence of any carbon source (black triangles) in AB minimum media.
