# Supplementary material for: A Pyranose-2-Phosphate Motif Is Responsible for Both Antibiotic Import and Quorum-Sensing Regulation in Agrobacterium tumefaciens
Source: PLoS Pathog. 2015 Aug 5;11(8):e1005071. doi: 10.1371/journal.ppat.1005071 (PMC4526662; doi:10.1371/journal.ppat.1005071)
Supplement: S4 Fig — Measures were done in triplicates. (PDF) [file ppat.1005071.s004.pdf]

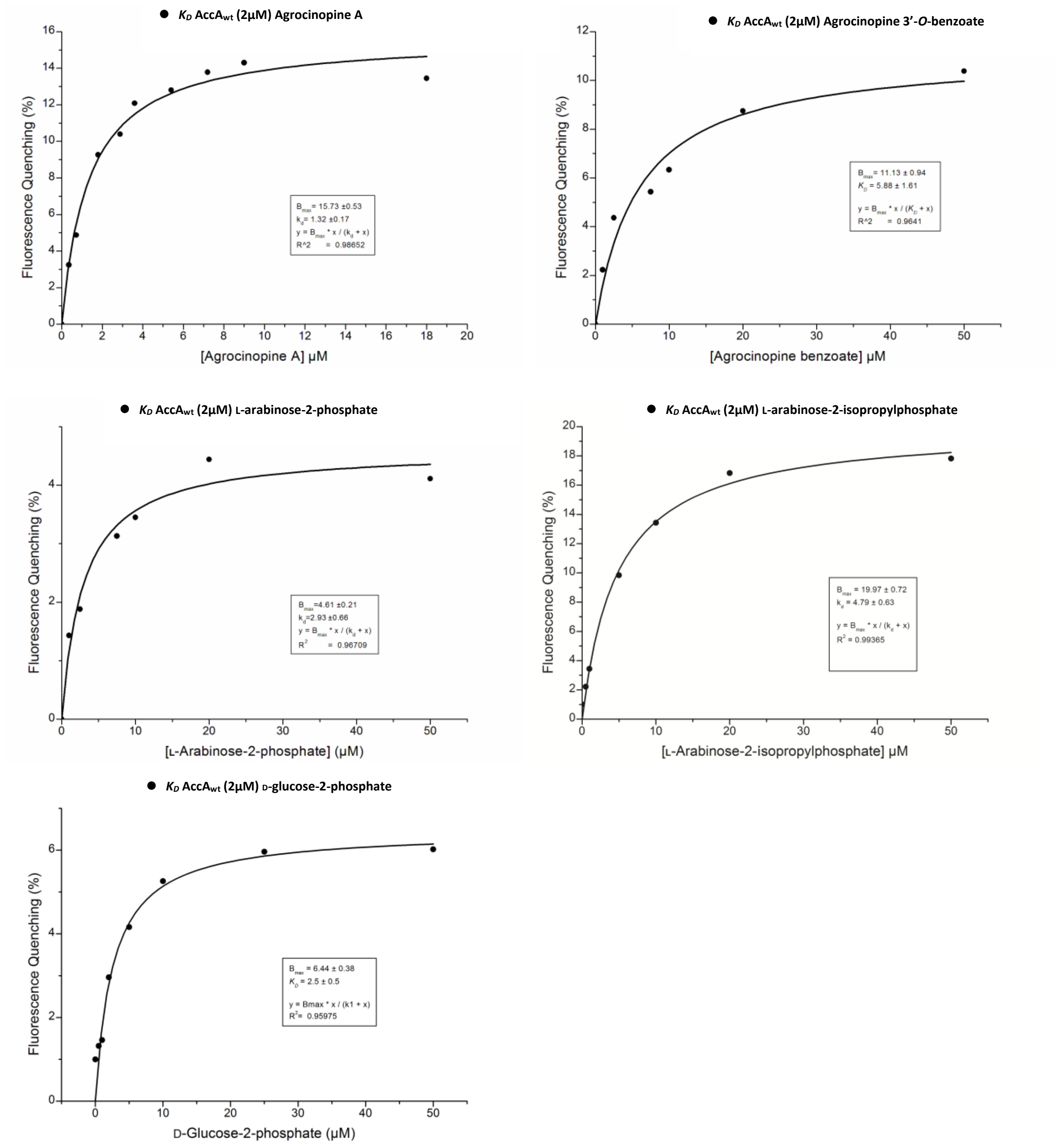

S4 Fig AccA fluorescence monitoring upon titration with each ligand and fit (solid line) to a single binding model using Origin. Measures were done in triplicates.
