# Supplementary material for: A Pyranose-2-Phosphate Motif Is Responsible for Both Antibiotic Import and Quorum-Sensing Regulation in Agrobacterium tumefaciens
Source: PLoS Pathog. 2015 Aug 5;11(8):e1005071. doi: 10.1371/journal.ppat.1005071 (PMC4526662; doi:10.1371/journal.ppat.1005071)
Supplement: S5 Fig — The top panels show heat differences upon injection of ligand and lower panels show integrated heats of injection and the best fit (solid line) to a single binding model using Microcal Origin. (PDF) [file ppat.1005071.s005.pdf]

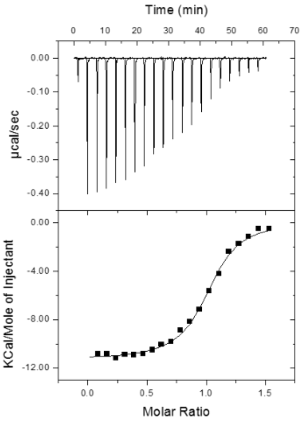

Agrocinopine A

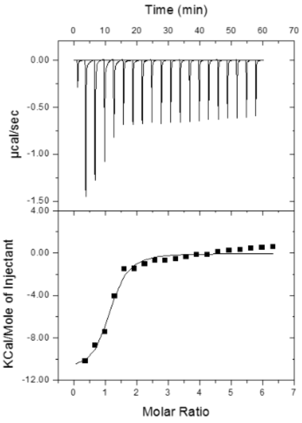

Agrocin 84

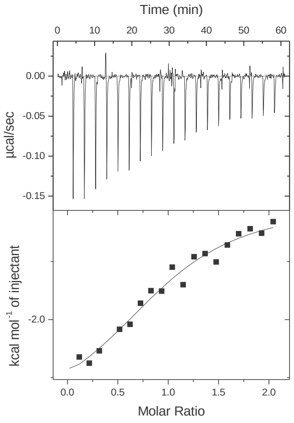

Agrocinopine 3’-*O*-benzoate

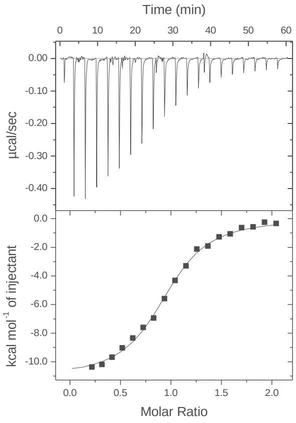

L-arabinose-2-phosphate

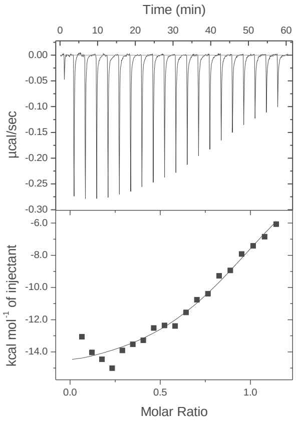

L-arabinose-2-isopropylphosphate

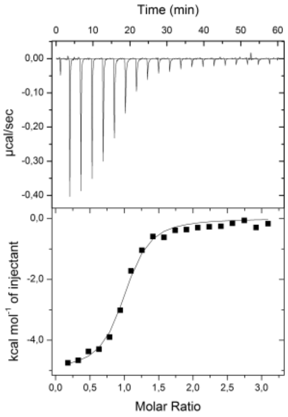

D-glucose-2-phosphate

S5 Fig AccA microcalorimetry measurements.The top panels show heat differences upon injection of ligand and lower panels show integrated heats of injection and the best fit (solid line) to a single binding model using Microcal Origin.
