# Supplementary material for: A Pyranose-2-Phosphate Motif Is Responsible for Both Antibiotic Import and Quorum-Sensing Regulation in Agrobacterium tumefaciens
Source: PLoS Pathog. 2015 Aug 5;11(8):e1005071. doi: 10.1371/journal.ppat.1005071 (PMC4526662; doi:10.1371/journal.ppat.1005071)
Supplement: S6 Fig — (a). between AccA (grey)-agrocinopine A and DPP (green)-GL complexes. Agrocinopine A and GL are shown in cyan and orange, respectively. The loop region 402–414 in AccA shown in green corresponds to the helix 383–399 from DPP shown in grey. (b) between AccA (grey)-agrocinopine A and DPP (green)-GL complexes. Agrocinopine A and GL are shown in cyan and orange, respectively. The two tryptophans gate (Trp178 and Trp423) and the loop 372–378 from AccA are represented in red while the corresponding residues (Met152 and Asp408) and the loop 351–359 in DPP are in deep blue. (PDF) [file ppat.1005071.s006.pdf]

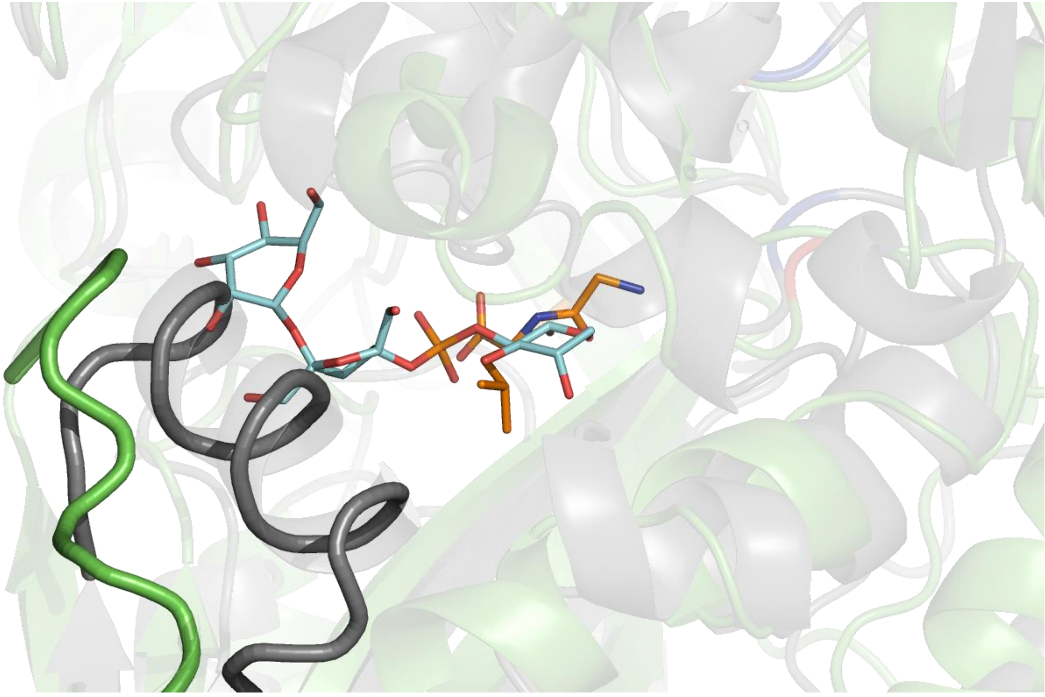

S6a Fig Structural comparison of the ligand binding site between AccA (grey)-Agrocinopine A and DPP (green)-GL complexes.
Agrocinopine A and GL are shown in cyan and orange, respectively. The loop region 402-414 in AccA shown in green corresponds to the helix 383-399 in DPP shown in grey.

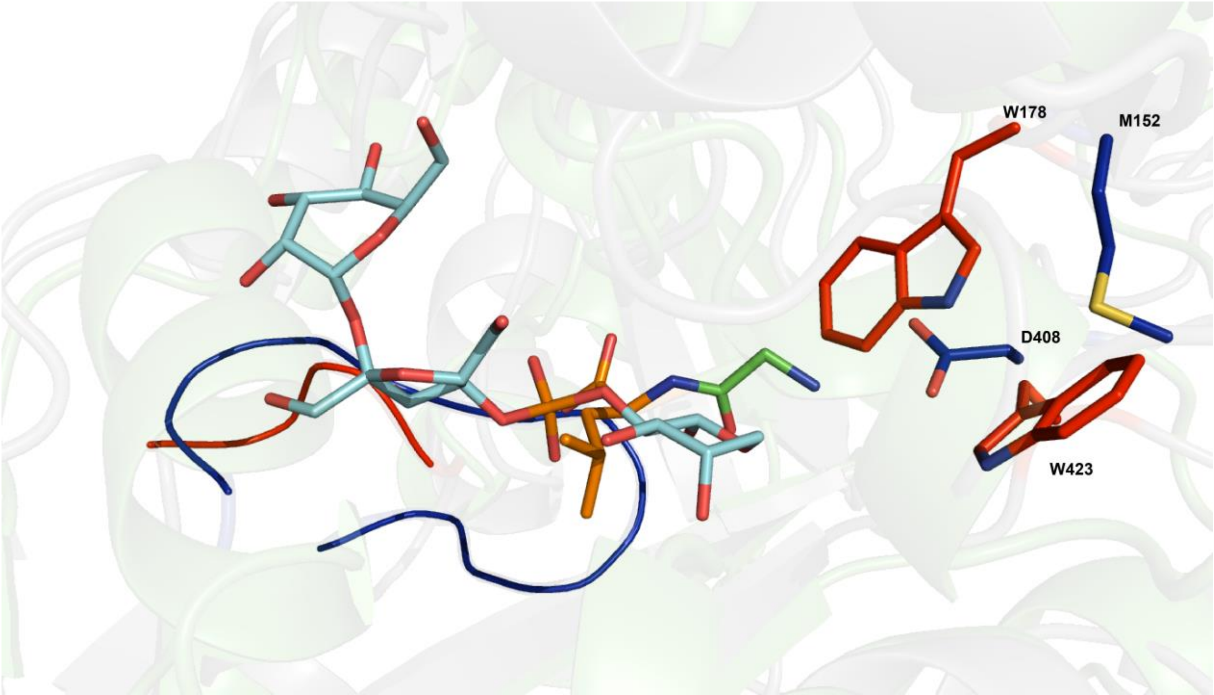

S6b Fig Structural comparison of the ligand binding site between AccA (grey)-Agrocinopine A and DPP (green)-GL complexes.
Agrocinopine A and GL are shown in cyan and orange, respectively. The two tryptophans gate (Trp178 and Trp423) and the loop 372-378 from AccA are represented in red while the corresponding residues (Met152 and Asp408) and the loop 351-359 in DPP are in deep blue.
