# Supplementary material for: A Pyranose-2-Phosphate Motif Is Responsible for Both Antibiotic Import and Quorum-Sensing Regulation in Agrobacterium tumefaciens
Source: PLoS Pathog. 2015 Aug 5;11(8):e1005071. doi: 10.1371/journal.ppat.1005071 (PMC4526662; doi:10.1371/journal.ppat.1005071)
Supplement: S7 Fig — tmCBP (PDB ID 2O7I & 4JSO) and AccA are colored in green and slate respectively. Agrocinopine A (yellow), cellobiose (light blue) and laminaripentaose (green) are represented as sticks. (PDF) [file ppat.1005071.s007.pdf]

a b

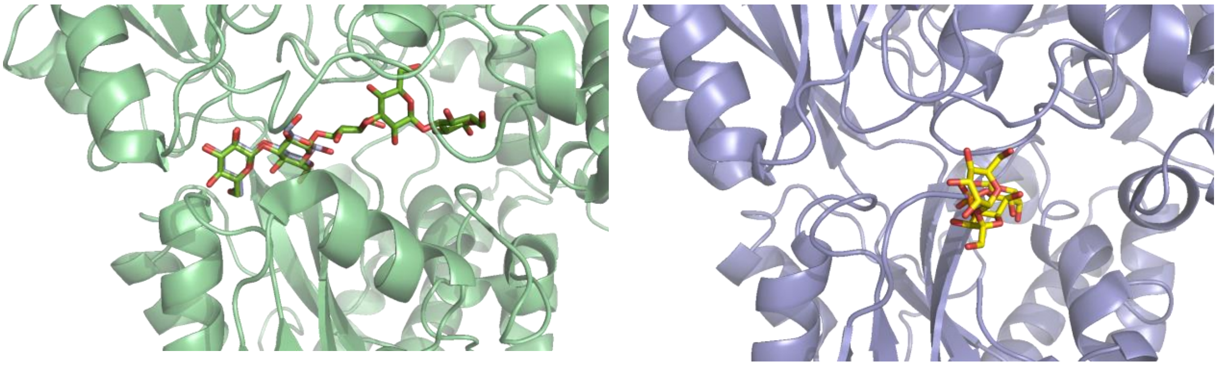

S7 Fig Ligand binding sites of tmCBP (a) and AccA (b). TmCBP (PDB ID 2O7I & 4JSO) and AccA are colored in green and slate respectively. Agrocinopine A (yellow), cellobiose (light blue) and laminaripentaose (green) are represented as sticks.
