# Supplementary material for: A Pyranose-2-Phosphate Motif Is Responsible for Both Antibiotic Import and Quorum-Sensing Regulation in Agrobacterium tumefaciens
Source: PLoS Pathog. 2015 Aug 5;11(8):e1005071. doi: 10.1371/journal.ppat.1005071 (PMC4526662; doi:10.1371/journal.ppat.1005071)
Supplement: S1 Table — The K D values were obtained using Microcal Origin. and fitting to a one binding site model using the following equation: f = ΔFluorescencemax*abs(x)/(K D+abs(x)). ND: no signal detected. (PDF) [file ppat.1005071.s008.pdf]

| | Agrocinopine A | Agrocin 84 | Agrocinopine 3’-*O*-benzoate | L-Arabinose-2-isopropylphosphate | L-Arabinose-2-phosphate | D-Glucose-2-phosphate |
|---|---|---|---|---|---|---|
| $K_D$ (µM) | 1.32 ± 0.17 | ND | 5.88 ± 1.6 | 4.79 ± 0.63 | 2.93 ± 0.66 | 2.50 ± 0.5 |

**S1 Table** Autofluorescence affinity results. The $K_D$ values were obtained using Microcal Origin. and fitting to a one binding site model using the following equation: $f=\Delta Fluorescence_{max}*abs(x)/(K_D+abs(x))$. ND: no signal detected.
