# Supplementary material for: A Pyranose-2-Phosphate Motif Is Responsible for Both Antibiotic Import and Quorum-Sensing Regulation in Agrobacterium tumefaciens
Source: PLoS Pathog. 2015 Aug 5;11(8):e1005071. doi: 10.1371/journal.ppat.1005071 (PMC4526662; doi:10.1371/journal.ppat.1005071)
Supplement: S2 Table — The values were obtained using Microcal Origin. and fitting to a one binding site model. No signal detected for L-arabinose and D-glucose. (PDF) [file ppat.1005071.s009.pdf]

	Agrocinopine A	Agrocin 84	Agrocinopine 3'-*O*-benzoate	L-Arabinose-2-isopropylphosphate	L-Arabinose-2-phosphate	D-glucose-2-phosphate
K_D (µM)	0.3 ± 0.03	1.5 ± 0.41	7.5 ± 2.2	2.2 ± 0.58	1.33 ± 0.12	1.16 ± 0.22
N	1 ± 0.006	0.8 ± 0.043	0.99 ± 0.06	1.08 ±0.03	0.95 ± 0.01	0,970 ± 0,01
K	2.99E6 ± 2.9E5	6.83E5 ± 1.8E5	1.33E5 ± 3.61E4	4.72E5 ± 1.2E5	7.54E5 ±6.5E4	8,61E5 ± 1,6
ΔH (cal/mol)	-1.12E4 ± 98	-4.37E4 ± 3.8E3	-3.7E3 ± 374	-1.56E4 ± 493	-1.11E4 ± 168	-4.5E3 ± 123
TΔS (cal/mol/K)	-2.6E3 ±	-3.6E4	3.2E3	-8E3	-3.2E3	-2.9E3
ΔG (cal/mol)	-8.6E3	-7.7E3	-6.9E3	-7.6E3	-7.9E3	-7.4E3

S2 Table Microcalorimetry results. The values were obtained using Microcal Origin. and fitting to a one binding site model. No signal detected for L-arabinose and D-glucose.
