# Supplementary material for: A Pyranose-2-Phosphate Motif Is Responsible for Both Antibiotic Import and Quorum-Sensing Regulation in Agrobacterium tumefaciens
Source: PLoS Pathog. 2015 Aug 5;11(8):e1005071. doi: 10.1371/journal.ppat.1005071 (PMC4526662; doi:10.1371/journal.ppat.1005071)
Supplement: S1 Text — Chemical synthesis and characterisation of synthetized molecules are described. (PDF) [file ppat.1005071.s010.pdf]

General Methods

General Methods for agrocinopine A, agrocinopine 3’-*O*-benzoate, L-arabinose-2-phosphate, L-arabinose-2-isopropylphosphate and D-glucose-2-phosphate synthesis

All solvents and reagents were purified by standard techniques or purchased from Sigma-Aldrich (dichloromethane, CH_3CN, DMF, MeOH, triethylamine and pyridine). Organic solutions were dried over anhydrous sodium sulfate. The reactions were performed under a constant flow of nitrogen. The reactions were monitored by t.l.c. on Silica Gel 60 F254 (Merck) and detection was carried out by UV light (254 nm) and/or and/or by spraying with a solution of 10% H_2SO_4 in EtOH. Silica gel (Kieselgel 60, 70–230 mesh ASTM, Merck) was used for column chromatography. The ^{1}H NMR (300 MHz or 400 MHz) and ^{13}C NMR (75 MHz or 100 MHz) spectra were recorded with Bruker ALS300, DRX300, and DRX400 spectrometers. Chemical shifts are given in ppm. Coupling constants are expressed in Hertz and splitting pattern abbreviations are: s, singlet; d, doublet; t, triplet; q, quartet; quint, quintuplet; m, multiplet; M, massif, p, pseudo; br, broad. Multiplicity (^{13}C NMR) was determined by DEPT sequences. High resolution mass spectra were obtained using an electro spray technique, in positive or negative mode with a ThermoFinnigan MAT 95 XL spectrometer. Optical rotations were measured with a Perkin–Elmer 343 polarimeter at 20°C (589 nm, sodium D line).

(1-*O*-benzyl-3,4-isopropylidene-β-L-arabinopyranos-2-yl)-(benzyl)-(isopropylamino)-phosphine (3).

To a solution of tetrazole (0.45 mol/L in acetonitrile, 5 mL) in anhydrous CH_2Cl_2 (5 mL) under argon was added diisopropylamine (0.37 mL). After stirring for 5 minutes, a solution of benzyl- 3,4-O- isopropylidene-β-L-Arabinose **1**[19] (0.92 g, 3.28 mmols) and benzyloxybis(diisopropylamino)phosphine **2**[20,21] in CH_2Cl_2 (25 mL) was added at 0°C to the formed salt. The mixture was warmed to room temperature and the stirring continued 2 h. After filtration and evaporation on the solvent the product was purified by column chromatography (EtOAc/pentane/Et_3N: 2/8/0.5 as eluant) to give **3** (1.39 g, 79%) as a colorless oil. **^{1}H-NMR** (300 MHz, $CDCl_3$): 7.0-7.5 (m, 10H, 2xPh); 4.94 (dd, 1H, ^{3}J = 3.33 Hz, H_1); 4.61 (dd, 2H, ^{3}J = 12Hz, ^{2}J = 54Hz, CH-O-<u>CH</u>$_2$-Ph); 4.48-4.82 (m, 2H, P-CH_2-Ph), 4.22-4.39 (m, 2H, H_3, H4); 3.89-4.08 (m, 3H, H_5, H_2); 3.67 (m, 2H, N-<u>CH</u>-CH_3); 1.3, 1.51 (2s, 6H, C-<u>CH</u>$_3$); 1.15-1.24 (4s, 12H, CH-<u>CH</u>$_3$). **^{13}C-NMR** (75 MHz, $CDCl_3$): 126-140

(2xPh); 108.9 C-CH_3); 98.01 (C_1); 75.6 (C_3); 73.8 (C_4); 73 (C_2); 69.8 (CH-C̲H_2-Ph); 65.3 (P-CH_2-Ph); 59.3 (C_5); 43.3 (C̲H-CH_3); 28.40.; 26.5; 24.75 and 24.65 (CH-C̲H_3); 11.8 (C-CH_3). **^{31}P-NMR** (300 MHz, $CDCl_3$): 150.32 and 149.05. **[α]D** = +92.6 (*c*1.0, CH_2Cl_2). **HRMS m/z (ESI)**: Calcd. for $C_{28}H_{41}NO_6P$. $(M+H)^+$: 518.2671. Found: 518.2666.

(3,6′-Di-*O*-acetyl-3′-*O*-benzoyl-2,1′:4,6-di-*O*-isopropylidene-sucrose)-4′-(1-*O*-benzyl-3,4-isopropylidene-β-L-arabinopyranos-2-yl)-(benzyl) phosphine (5).

To a solution of the protected sucrose **4**[22] (1.2 g, 1.97 mmol) and the arabinophosphine **3** (1.3 g, 2.5 mmol) in anhydrous CH_3CN (15 mL) was added at room temperature a solution of tetrazole (0.45 M in CH_3CN, 4.3 mL). The reaction mixture was stirred 2h. After filtration and evaporation of the solvent the product was purified by column chromatography (ether/pentane/Et_3N: 1/1/ as eluant in the presence of few drops of Et_3N) to give **5** (1.85 g, 91% yield) as a yellowish syrup. **^{1}H-NMR** (300 MHz, CDCl3):7.43-8.12(m, 5H, Bz); 7.09-7.38 (m, 10H, 2xPh); 6.085 (2xd, ^3J = 1.65 Hz, 1H; G1); 5.36 (2xd, ^3J = 5 Hz, 1H, G3), 5.06 (2xd, 1H, ^3J = 9Hz, A1); 4.73-4.95 (m, 3H, F3, C̲H̲$_2$-Ph); 4.66 (dd, 2H, ^3J = 6Hz, ^2J = 12 Hz, C̲H̲$_2$-Ph); 4.44 (d, 2H, ^3J = 9Hz, ^2J = 12 Hz, F6); 4.37 (d, 1H, ^3J = 3Hz, F5); 4.33 (dd, 1H, ^3J = 3 Hz, 6 Hz, F4); 4.16-4.28 (m, 3H, A3, A4, A2); 4.09 (2xd, 1H, ^2J = 12 Hz, F1a); 3.92-3.94 (m, 2H, A5, G6a); 3.87 (dd, 1H, ^3J = 3 Hz, 15 Hz, G5); 3.75-3.82 (m, H, G2); 3.56-3.60 (m, 2H, G6b, G4); 3.53 (d, 1H, ^2J = 12Hz, F1b); 1.20-1.45 (6xs, 18H, C-C̲H_3). **^{13}C-NMR** (75 MHz, CDCl3): 170.73 et 169.76 (C̲O-CH_3); 165.94 (C̲O-Ph); 127.26-137.32(Ph); 109.20 (C̲-CH_3, A); 104.6 (F2); 99.6 et 101.4 (C̲-CH_3; G et F); 97.28 (A1); 91.6 (G1); 77.36 (F3); 74.81 (A2); 73.76 (G3); 72.78 (G5); 71.79 (G2); 71.52 (A4); 70.85 (A3+G4); 69.61 (P-CH-C̲H_2-Ph); 67.86 (O-C̲H_2-Ph,A); 66.15 (F6); 64.15 (F1+A5); 59.18 (G6); 25.59 et 29.15 (CO-CH3); 28.31; 26.38; 24.01 et 21.04 (CH-C̲H_3,G et F); 11.73 (C-C̲H_3, A). **^{31}P-HMR**: (300 MHz, CDCl3): 139.8 and 139.43. **[α]D** = +70.2 (*c* 1.0, CH_2Cl_2). **HRMS m/z (ESI):** Calcd. for $C_{51}H_{64}O_{20}P$. $(M+H)^+$: 1027.3729. Found: 1027.3739.

3,6′-Di-*O*-acetyl-3′-*O*-benzoyl-2,1′:4,6-di-*O*-isopropylidene-sucrose)-4′-(1-*O*-benzyl-3,4-isopropylidene-β-L-arabinopyranos-2-yl)-(benzyl)-phosphate (6).

To a solution of **5** (0.49 g, 0.477 mmol) in CH_2Cl_2 (5 mL) was added *t*BuOOH (5.5 M in decane, 104 μL) at 0°C and the solution obtained was stirred for 2 h. The reaction mixture was concentrated and purification of the residue by silica gel flash column chromatography (EtOAc/pentane/Et_3N: 3/7/0.2), gave **6** as a yellowish syrup (0.46 g, 92%). **^{1}H-NMR** (300 MHz, $CDCl_3$): 7.45-8.15 (m, 5H, CO-P̲h̲); 7.2-7.34 (m, 10H, CH_2-P̲h̲); 6.05 (2xd, 1H, ^3J =

3Hz, G1); 5.35 (2xd, 1H, ^3J = 3Hz, G3); 4.93-5.11 (m, 4H, A1, 2xCH2-Ph); 4.68 (dd, 1H, F3); 4.47 (dd, 2H, ^3J = 12 Hz, ^2J = 30 Hz, F6); 4.37-4.54 (m, 3H, F6, F5); 4.35 (dd, 1H, ^3J = 9 and 12Hz, F4); 4.19-4.29 (m, 3H, A3, A4, A2); 4.17 (d, 1H, ^2J = 12Hz, F1a); 3.92-3.95 (m, 2H, A5, G6a); 3.85 (dd, 1H, ^3J = 6 et 15 Hz, G5); 3.74-3.79 (m, 1H, G2); 3.51-3.61 (m, 3H, G6b, G4, F1b). **^{13}C-NMR** (75 MHz, CDCl3): 170.6 et 169.7 (CO-CH_3); 165.6 (CO-Ph); 127.8-137 (Ph); 109.5 (C-CH_3, A); 104.9 (F2); 99.4 et 101.4 (C-CH_3; G et F); 96 (A1); 91.7 (G1); 81.4 (F4); 78 (F5); 78.7 (F3); 76.9 (A2); 77 (G3); 74.7 (G5) ; 73.7 (G2); 71.7 (A4); 71.3 (G4); 70.7 (A3); 69.9 (P-CH2-Ph,A); 69.6 (CH-CH2-Ph, A); 66.2 (F6); 64.2 (F1); 61.9 (G6); 58.8 (A5); 25.6 et 29 (CO-CH3); 28.1; 26; 24 et 21 (CH_3, G et F); 19 (C-CH_3, A). **^{31}P-HMR** (300 MHz, $CDCl_3$): -2.2 and -2.3. **HRMS m/z (ESI):** Calcd. for $C_{51}H_{63}NaO_{21}P$. $(M+Na)^+$: 1065.3497. Found: 1065.3492.

(3,6′-Di-*O*-acetyl-3′-*O*-benzoyl-sucrose)-4′-(1-*O*-benzyl-β-L-arabinopyranos-2-yl)-(benzyl)- phosphate (7).

A solution of **6** (0.46 g, 0.42 mmol) in 60% aqueous acetic acid (45 mL) was heated to 50°C for 30 min, then concentrated. Purification of the residue by silica gel flash column chromatography (CH_2Cl_2 / MeOH 95:5, *V*/*V*) gave the pure partially protected phosphate **7** as colorless syrup (0.205 g, 53 % yield). **^{1}H-NMR** (500 MHz, CD_3OD): 7.49– 8.18 (m, 5H, CO-Ph); 7.14 – 7.40 (m, 10H, 2xBn); 6.00 (d, 0.5H, ^3J = 7.4Hz, F_3); 5.98 (d, 0.5H, ^3J = 7.8Hz, F3′); 5.475 (d, 0.5H, ^2J =3.6, G_1); 5.46 (d, 0.5H, ^3J =3.55 Hz, G_1'); 5.36 (dd, 1H, ^3J = 4.25 et 7.3 Hz, G_3); 5.31 (dd, 0.5H, ^3J = 9.7 et 19.45, F_4); 5.187 (dd; 0.5H, ^3J = 7.55 et 15.15 Hz, F_4'); 5.12 (d, 0.5H, ^3J = 3.45 Hz, A_1); 5.03 (d, 0.5H, ^3J = 3.5 Hz, A_1'); 4.90-5.00 (m, 2H, $POCH_2Ph$); 4.74 (d, 0.5H, ^2J = 12.1 Hz, CH_2Ph); 4.59 (m, 1H, A_2); 4.447 – 4.553 (m, 3H, F_{6a}, F_{6b} et CH_2-Ph); 4.369 – 4.10 (m, 1H, CH_2-Ph et F_5); 4.30 (ddd, ^3J = 3.95, 7.3 Hz, ^2J=14.7 Hz, 0.5H, F_5'); 4.03 (dd, 0.5H, ^3J = 6.25 et 9.6 Hz, A_3); 3.38- 3.99 (m, 5H, G_{6a}; A_5; A_3'; A_4; G_5'); 3.76 - 3.80 (m, 2H, G_{6b}; F_{1a}); 3.59-3.69 (m, 3.5H, F_{1b}, G_4, G_2, G_5'); 2.14 (s, 3H, $COCH_3$); 2.14 (s, 3H, $COCH_3$); 2.075 (s, 3H, $COCH_3$); **^{13}C-NMR** (125 MHz, CD_3OD): 172.73; 172.77 (CO-G_3); 172.61; 172.62 (CO-F_6); 167.13; 167.16 (CO-Ph); 128.78- 138.74 (Ph); 105.1; 105.3 (F_2); 97.8; 97.7; 97.6; 97.7 (A_1); 93.63; 93.8 (G_1); 80.7; 80.8; 80.9; 81 (F_4); 79.4; 79.6; 79.7; 79.8 (F_5); 78.10; 78.13; 78.34; 78.4 (F_3); 77.7; 77.76; 77.8 (A_2); 76.9; 76.9 (G_3); 74.5; 74.7 (G_5); 71.1 – 71.3 (G_2 + $POCH_2Bn$); 70.09; 71.04 (A_4); 70.35; 70.44 (A(CH_2Bn)); 69.08; 69.17 (G_4); 68.8; 68.9; 69; 69.1 (A_3); 66 ; 66.2 (F_6); 64 ; 64.2 (F_1+A_5); 61.8; 61.9 (G_6); 20.7-21.2 (2x(CO-CH_3)). **^{31}P-HMR** (500 MHz, CD_3OD): -2.47 and -2.82. **$[\alpha]_D$** = +74.6 (*c* 0.5 CH_3OH). **HRMS m/z (ESI)**: Calcd. for $C_{42}H_{51}O_{21}PNa$: $(M+Na)^+$: 945.2558. Found: 945.2553.

(3,6′-*O*-diacetyl-3′-*O*-benzoyl-sucrose)-4′-(β-L-arabinopyranos-2-yl)-phosphate (8).

Pd/C (110 mg) was added to a solution of phosphate **7** (0.23 g, 0.249 mmol) in ethanol (10 mL) and the mixture stirred under an H_2 atmosphere for 24 h at room temperature. The solution was then filtered through celite and concentrated in vacuo to give **8** as colorless syrup (155 mg, 83% yield). **8:** ^{1}**H-NMR** (400 MHz, CD_3OD): 7.48 – 8.12 (m; 5H; CO-Ph); 5.82(s_{br}, 1H, F3); 5.39 (m, 1H, G1); 5.26 (dd, 1H, ^{3}J = 9.6Hz, G3); 5.16 (dd, 1H, ^{3}J = 12Hz, F4); 5.04 (m, 1H, A1); 4.38 – 4.61 (m, 3H, F4, F5, G5); 3.48-4.01 (m, 13H, A3, G6, F6, A5, F1, A3, G4, A4, G2, A2); 2.05 (s, 6H, 2xCOCH_3). ^{13}**C-NMR** (100 MHz, CD_3OD): 172.76; 172.84 (2xCO-CH_3); 167.01 (CO-Ph); 127.41-132.06 (Ph); 105.61 (F2); 93.65 (G1); 80.99 (F4); 78.99 (F5); 78.71 (F3); 77.77 (A2); 76.63 (G3); 74.58(G5); 73.08 (G2); 72.29 (A4); 69.18(G4); 69.07(A3); 64.32 (F6); 61.25 - 6.27 (F1+A5); 59.67(G6); 18.53; 18.87 (2*CO-CH_3). ^{13}**P-NMR** (400 MHz, CD_3OD): -0.09 (s_{br}). **HRMS m/z (ESI):** Calcd. for $C_{28}H_{38}O_{21}P$**.** (M-H): 741.1643. Found: 741.1723 pour $[\alpha]_D$ = +30.1 (*c* 1.13 CH_3OH).

Sucrose-4′-(α,β-L-arabinopyranos-2-yl)-phosphate (9, agrocinopine A).

To a solution of phosphate **8** (46 mg, 0.062 mmol) in dry MeOH was added K_2CO_3 (33 mg, 0.239 mmol) and the mixture was stirred at RT for 2 h. After filtration and concentration the residue was dissolved in water (1 mL) and applied to column of DEAE-Sepharose. The column was eluted with gradient (0 and 0.5 M) of sodium chloride. Fractions containing **9** were concentrated and the residue was applied to Bio-Gel column (H_2O as eluent) followed by filtration through a Sep-Pak™ column to give pure **9** (14 mg, 34% yield) as a colorless syrup. ^{1}**H-HMR** (500 MHz, D2O): 5.38-5.48 (m, 2H, G1 et Aβ1); 4.77 (dd, 1H, ^{3}J =5.25, 16 Hz, Aα1); 4.54 (d, ^{3}J = 7.85 Hz, 1H, F4); 4.42 (d, ^{3}J = 8,15 Hz, 1H, F3); 4.29-4.36 (dd, 1H, Aβ2); 3.78-4.13 (m, 12H, Aα2, Aβ4, F5, Aβ3, G5, G6a, Aα5, Aα5b, Aα5a, Aβ5a, F6, G3) ; 3.62-3.70 (m, 2H, F1 et Aβ5b); 3.55-3.58 (dd, J 3.35, 9.9 Hz, 1H, G2); 3.49 (dd, ^{3}J 9.75 Hz, 1H, G4). ^{13}**C-HMR** (125 MHz, D_2O): 104 (F2); 92.3 (A1); 91.4 (G1); 81.1 (F4); 78.3 (F5); 75.7 (F3); 73.6 (A2); 72.5 (G3); 72.3 (G5); 71.1 (G2); 69.2 (A4); 68.5 (G4); 67.8 (A3); 65.3 (F6); 62.3 (A5); 61.2 (F1); 60 (G6). ^{31}**P-HMR** (300 MHz, D_2O) :-0.3 et -0.47. $[\alpha]_D$ = +33.4 (*c* 0.09, H_2O). **HRMS m/z (ESI)**: Calcd. for $C_{17}H_{30}O_{18}P$. $(M -H)^{-+}$: 553.1170 Found: 553.1163. Alternatively, the potassium salt form of **9** can be obtained: to a solution of phosphate **8** (220 mg, 0.296 mmol) in anhydrous MeOH (20 mL) was added K_2CO_3 (145 mg, 1.05 mmol) and the mixture was stirred at RT for 2 h. After elimination of K_2CO_3 by filtration and evaporation of the solvent, the residue was crystallized from MeOH (3 mL) to give pure agrocinopine

potassium salt (110 mg, 68%) as a pale yellowish solid. **[α]$_D$** = +43.4° (C=0.6, H_2O). **HRMS m/z (ESI)**: Calcd. for $C_{17}H_{30}KNaO_{18}P$. $(M + Na)^+$: 615.0705 Found: 615.0699.

(3′-*O*-Benzoyl-sucrose)-4′-(α,β-L-arabinopyranos-2-yl)-phosphate (10, agrocinopine 3’-***O*-benzoate)**

The phosphate **8** (60 mg, 0.076 mmol) was dissolved in methanol (6 mL) and 1M methanolic sodium methoxide (50 uL) added at 0°C. The mixture was allowed at room temperature and neutralized after 30 min with dry ice and then concentrated. The residue was purified following the procedure described for the phosphate **9** to give a mixture (35 mg, >60% yield) of **9** and **10** as major product (>80%). Alternatively, to a solution of phosphate **8** (24 mg, 0.032 mmol) in methanol (5 mL) at 0°C was added 0.1M methanolic sodium methoxide (300 μL). After stirring 30 min at 0°C, addition of a spatula of Amberlite resin IR 120 H^+ and filtration, concentration of the solvent gave the phosphate **10** (16 mg, 76%) as a pale yellow syrup. **10**: **^{1}H-NMR** (400 MHz, CD_3OD): 8.16 (2xd, J = 10 Hz, 2H, Ph); 7.45-7.77 (m; 3H; Ph); 5.85 (d; 0.5H; ^3J = 6.8Hz; F3); 5.89 (d; 0.5H; ^3J = 6.4 Hz; F3′); 5.42 (m; 1H; G1); 5.36 (dd; 1H; ^3J = 4.25; 7.3Hz; G3); 5.12 (dd; 1H; ^3J = 9.65 et 20.35Hz; F4); 4.98 (m; 1H; A1); 4.29 – 4.61 (m; 3H; F4; F5; G5); 3.54-4.01 (m; 13H; A3; G6; F6; A5; F1; A3; G4; A4; G2; A2). **^{13}C-NMR** (100 MHz, CD_3OD): 172.9 (CO-G3); 167.32 (CO-Ph); 129.60, 129.69, 129.79, 130.92, 130,98, 131,28, 134.28 and 134.35 (Ph); 109.24 (A1); 105.53 (F2); 93.67 (G1); 85.21 (F4); 83.60 (F5); 78.66 (F3); 76.93 (A2+F4); 74.48 (G5+G3); 71.37 (G2); 69.08-69.18 (G4+A4+A3); 65.94 (F6); 63.54 - 64.24 (F1+A5); 63.02 (G6). **^{31}P-HMR** (400 MHz, CD_3OD): 0.98 and 0.23. **MS m/z (ESI)**: Calcd. for $C_{24}H_{34}O_{19}P$. $(M-H)^-$: 657.1432 Found: 657.1419.

(1-*O*-Benzyl-3,4-isopropylidene-β-L-arabinopyranos-2-yl)-(dibenzyl) phosphine (11).

To a solution of arabinophosphine **3** (1.07 g, 2.07 mmol) and benzyl alcohol (0.27g, 2.49 mmol) in anhydrous CH_3CN (8 mL) was added at room temperature a solution of tetrazole (0.45 M in CH_3CN, 1.8 mL). The reaction mixture was stirred 30 minutes followed by addition of CH_2Cl_2 (50 mL). The solution was then washed with sat aq. $NaHCO_3$, dried with sodium sulfate and the solvent evaporated. The product was purified by column chromatography (ether/pentane/Et_3N: 1/1/ as eluent in the presence of few drops of Et_3N) to give **11** (0.91 g, 84% yield) as a colorless oil. **^{1}H-NMR** (300 MHz, $CDCl_3$): 7.26-7.33 (m, 15H, 3xPh); 4.83-4.89 (m, 5H, 2xPOC̲H_2Bn, A1); 4.44-4.73 (d, 2H, ^2J = 12Hz, CHOPC̲H_2Bn); 4.24-4.39 (m, 2H, A3, A4); 4.12 (dd, ^3J = 6Hz, A2); 3.99 (s, 2H, A5). 1.35 et

1.48 (2xCH_3) ; **^{13}C-NMR** (75 MHz, $CDCl_3$): 127.45, 127.67, 127.74, 127.92, 127.94, 128.46, 128.50 and 128.53 (3xPh); 109.24 ($\underline{C}$-CH_3); 97.51 (A1); 77.36 (A3); 75.09 (A2); 73.88 (A4); 69.62 (CH-O-$\underline{C}H_2$-Ph); 64.26 (2xP-O-$\underline{C}H_2$-Ph); 59.24(A5); 28.28 et 26.25 (C-$\underline{C}H_3$) **^{31}P-HMR**: (300 MHz, CDCl3): 139.50. **$[\alpha]_D$** = +136.6 (*c* 0.6 CH_2Cl_2). **HRMS m/z (ESI):** Calcd. for $C_{29}H_{33}O_7PNa$. $(M+Na)^+$: 547.1862. Found: 547.1856.

(1-*O*-Benzyl-3,4-isopropylidene-β-L-arabinopyranos-2-yl)-(dibenzyl)-phosphate (12).

Prepared following the same procedure as for the phosphate **6** with 0.9 g (1.7 mmol) scale of phosphine **11**. We obtain 1 g (100% yields) of **12** as colorless syrup. **^{1}H-NMR** (300 MHz, $CDCl_3$): 7.30-7.32 (m, 15H, 3xPh); 4.96-5.07 (m, 5H, 2xPO$\underline{C}H_2$Bn, A1); 4.45-4.72 (d, 2H, ^2J = 12Hz, CHOP$\underline{C}H_2$Bn); 4.23-4.25 (m, 3H, A2, A3, A4); 3.99(s, 2H, A5). 1.33 et 1.46 (2xCH_3); **^{13}C-NMR** (75 MHz, $CDCl_3$): 127.91, 128.04, 128.08, 128.52, 128.53, 128.57, 128.61 and 128.62 (3xPh); 109.55 ($\underline{C}$-CH_3); 96.42 (A1); 77.36 (A3); 74.23 (A2); 73.87 (A4); 69.96 (CH-O-$\underline{C}H_2$-Ph); 69.28 and 69.25 (2xP-O-$\underline{C}H_2$-Ph); 59.9(A5); 28.16 et 26.49 (C-$\underline{C}H_3$) **^{31}P-HMR**: (300 MHz, CDCl3): -1.50. **$[\alpha]_D$** = +135 (*c* 0.6 CH_2Cl_2). **HRMS m/z (ESI)**: Calcd. for $C_{29}H_{33}O_8PNa$. $(M+Na)^+$: 563.1811. Found: 563.1802. The D-enantiomer of intermediate **12** has been previously reported (Anastasi *et al.*, *Chem. Eur. J.* 2008 *14*, 2375-2388).

(1-*O*-Benzyl-β-L-arabinopyranos-2-yl)-(dibenzyl)-phosphate (13).

Compound **13** was prepared from **12** (0.927 g, 1.72 mmol) by using the procedure described for the preparation of **7**, yielding **13** (0.876 g, 93%) as white solid. **^{1}H-NMR** (300 MHz, CD_3OD): 7.26-7.33 (m, 15H; 3xPh); 5.10(d, ^3J =3Hz, 1H, A1); 4.96-5.07 (m, 4H, 2xPO$\underline{C}H_2$Bn); 4.42-4.73 (d, 2H, ^2J = 12Hz, CHOP$\underline{C}H_2$Bn); 4.57-4.60 (m, 1H, A2); 4.04 (dd, ^3J = 3Hz, 1H, A3); 3.89-3.90 (dd, ^2J = 12Hz, ^3J =3Hz, 2H, A5); 3.64 (dd, ^2J = 12Hz, ^3J =3Hz, 1H, A4). **^{13}C-NMR** (75 MHz, CD_3OD): 128.84, 129.07, 129.14, 129.43, 129.59 and 129.61 (3xPh); 98.11 (A1); 77.36 (A3); 77.27 (A2); 71.03 (A4); 70.90 (CH-O-$\underline{C}H_2$-Ph); 70.63 (2xP-O-$\underline{C}H_2$-Ph); 64.26(A5). **^{31}P-HMR**: (300 MHz, CD_3OD): -1.83. **$[\alpha]_D$** = +89 (*c* 1 CH_2Cl_2). **HRMS m/z (ESI)**: Calcd. for $C_{26}H_{29}O_8PNa$. $(M+Na^+)$: 523.1498. Found: 523.1492.

(L-Arabinopyranos-2-yl)-phosphate (14, arabinose-2-phosphate).

Compound **14** was obtained from **13** (0.876 g, 1.752 mmol) by using the procedure described for compound **4**, which gave the product as colorless oil (0.39 g, 97%). **^{1}H-NMR** (300 MHz, CD_3OD): 5.26 and 5.31 (2xs, 1H, A1); 4.42-4.62 (2d; ^3J = 3Hz, 1H, A2); 3.77- 4.04 (m, 4H, A3, A5, A4). **^{13}C-NMR** (75 MHz, CD_3OD):): 91.60 (A1) ; 75.93 (A3) ; 75.20 (A2); 71.03

(A4) ; 64.35(A5). ^{31}**P-HMR**: (300 MHz, CD_3OD): 0.35 and -0.77. **HRMS m/z (ESI):** Calcd. for $C_5H_{11}O_8PNa$. (M+Na$^+$). 253.0089 found: 253.0084. The D-enantiomer of intermediate **14** has been previously reported (Anastasi *et al.*, *Chem. Eur. J.* 2008 *14*, 2375-2388).

Isopropyl-(1-*O*-benzyl-3, 4-isopropylidene-β-L-arabinopyranos-2-yl)-(benzyl) phosphine (15).

Compound **15** was prepared from **14** (0.49 g, 0.948 mmol) with isopropanol (0.12 g, 1.9 mmol) by using the procedure described for the preparation of **11**, yielding the title compound **15** (0.22 g, 50%) as colorless liquid. ^{1}**H-NMR** (300 MHz, $CDCl_3$): 7.23-7.30 (m, 10H, 2xPh); 4.92 (d, 1H; ^3J = 3Hz; A1) ;4.80-4.83(m; 2H; PO$\underline{C}H_2$Bn) ; 4.66-4.70 (d, 2H, ^2J = 6Hz, CHOP$\underline{C}H_2$Bn), 4.21-4.50 (m, 4H, A2, A3, A4, CH); 3.96(s, 2H, A5). 1.32; 1.46 (2s, 6H, 2xCH_3); 1.48 (d, ^3J = 3Hz, 6H, 2xCH_3). ^{13}**C-NMR** (75 MHz, $CDCl_3$): 127.28, 127.59, 127.90, 127.97, 128.39, 128.43, 128.50, 128.55 (2xPh); 109.14 ($\underline{C}$-CH_3); 97.6 (A1) ; 77.38 (A3) ; 75.11 (A2) ; 73.88 (A4) ; 71.88 ($\underline{C}$H-CH_3); 69.64 (CH-O-$\underline{C}H_2$-Ph); 63.41 (P-O-$\underline{C}H_2$-Ph); 59.16(A5); 24.64, 26.49 et 28.39 (4xCH_3). ^{31}**P-HMR**: (300 MHz, $CDCl_3$): 139.5 et 139.06. **HRMS m/z (ESI)**: Calcd. for $C_{25}H_{34}O_7P$ (M+H)$^+$: 477.2042 Found: 477.2038.

Isopropyl-(1-*O*-benzyl-3, 4-isopropylidene-β-L-arabinopyranos-2-yl)-(benzyl)-phosphate (16).

The phosphate **16** was prepared from **15** (0.22 g, 0.462 mmol) by using the procedure described for the preparation of the phosphate **6**, yielding **16** (0.3 g, 100%) as colorless syrup.

^{1}**H-NMR** (300 MHz, $CDCl_3$): 7.23-7.27 (m; 10H; 2xPh) ; 5.14 (d, 1H, ^3J = 6Hz, A1); 4.98-5.09 (m, 2H , PO$\underline{C}H_2$Bn) ; 4.58-4.75 (m, 3H, CHOP$\underline{C}H_2$Bn, A2); 4.33-4.50 (m, 2H, A3, A4); 4.24 (m, 1H, CH), 3.95 (m, 2H, A5); 48 and 1.46 (2xs, 6H, 2xCH_3); 1.32 (d, ^3J = 6Hz, 6H, 2xCH_3). ^{13}**C-NMR** (75 MHz, $CDCl_3$): 127.73, 127.93, 128.01, 128.03, 128.37, 128.39, 128.56 and 128.58 (2xPh); 109.44 ($\underline{C}$-CH_3) ; 96.59 (A1); 77.36 (A3); 74.30 (A2); 74.21 (A4); 73.40 ($\underline{C}$H-CH_3); 69.96 (CH-O-$\underline{C}H_2$-Ph); 69.13 (P-O-$\underline{C}H_2$-Ph); 58.96(A5); 24.64, 26.49 et 28.39 (4xCH_3).^{31}**P-HMR**: (300 MHz, $CDCl_3$): -2.34 and -2.38. **HRMS m/z (ESI):** Calcd. for $C_{25}H_{34}O_8P$ (M+H)$^+$: 493.1991 Found: 493.1986.

Isopropyl-(1-*O*-benzyl-3-β-L-arabinopyranos-2-yl)-(benzyl)-phosphate (17).

The title compound was prepared from **16** (0.3 g, 0.61 mmol) by using the procedure described for the preparation of phosphate **7**, yielding **17** (0.178g, 64%) of colorless syrup. ^{1}**H-NMR** (300 MHz, CD_3OD): 7.27-7.36 (m, 10H, 2xPh); 5.12 (2xd, ^3J =3Hz, 1H, A1), 4.98-

5.07 (m, 2H, PO$\underline{C}H_2$Bn); 4.69-4.75 (d, 2H, ^2J = 6Hz, CHOP$\underline{C}H_2$Bn); 4.61-4.65(dd, ^3J = 3 Hz, 1H, A2); 4.54-4.59 (m, 1H, A3); 4.43-4.53 (m, 1H, CH); 3.88-4.02 (m, 2H, A5); 3.62-3.66 (m, 1H, A4); 1.34 (d, ^3J = 6Hz, 6H, 2xCH_3). **^{13}C-NMR** (75 MHz, CD_3OD): 128.84, 128.87, 128.97, 129.05, 129.06, 129.12, 129.42, 129.52, 129.58 and 129.60 (2xPh); 98.22 (A1) ; 77.03 (A3); 74.93 (A2); 71.02 (A4); 70.60 ($\underline{C}H$-CH_3); 69.96 (CH-O-$\underline{C}H_2$-Ph); 69.03 (P-O-$\underline{C}H_2$-Ph); 64.25(A5); 23.79 et 30.69 (2xCH_3). **^{31}P-HMR**: (300 MHz, CD_3OD): -2.57 et -2.65. **HRMS m/z (ESI):** Calcd. for $C_{22}H_{30}O_8P$. $(M+H)^+$: 453.1678. Found: 453.1673.

Isopropyl-(α,β-L-arabinopyranos-2-yl)-phosphate (18, arabinose-2-isopropylphosphate).

Compound 18 was prepared from 17 (0.178 g, 0.394 mmol scale) following the procedure described for phosphate 8, which gave the product as colorless syrup (0.105 g, 98%). **^{1}H-NMR** (300 MHz, CD3OD): 5.03 (2xd; 3J =3Hz, 1H, A1); 4.60-4.65 (dd, 3J = 3 Hz, 1H, A2); 4.52-4.57 (m, 1H, A3); 4.43-4.53 (m, 1H, CH); 3.88-4.02 (m, 2H, A5); 3.62-3.66 (m, 1H, A4). **^{13}C-NMR** (75 MHz, CD3OD): 92.4 (A1); 76.73 (A3); 74.80 (A2); 71.02 (A4); 70.60 (CH-CH3); 64.25(A5) ; 23.79 et 30.69 (2xCH3).**^{31}P-HMR**: (300 MHz, CD3OD): -1.36 and -2.38. **HRMS m/z (ESI)**: Calcd for C5H11O8P. (M-H+): 271.0583. Found: 271.0588.

Benzyl 3,4,6-tri-*O*-benzyl-α- D-glucopyranoside (**21**): For the synthesis of compound **21**, D-glucose was glycosylated[1] and benzylated leading to known compound **20** which gave analytical data identical to those reported in literature [1]. TIBAL (2.4 mL, 9.5 mmol) was added to a solution of compound **20** (1 g, 1.59 mmol) in toluene (10 mL) and the mixture was stirred for 60 h at 50°C. The reaction mixture was then cooled to 0°C and HCl 1 M was added drop wise. Ethyl acetate was added, the organic phase was collected and the aqueous phase was extracted twice with ethyl acetate. The organic phases were combined, dried over $MgSO_4$, filtered and evaporated. After purification by silica gel chromatography (petroleum ether/EtOAc 4:1), compound **21** (220 mg, 26%) was obtained as a colorless oil and gave analytical data identical to those reported in literature [2].

Benzyl 2-*O*-dibenzyl phosphate 3,4,6-tri-*O*-benzyl-α-D-glucopyranoside (**22**): *N,N*-diisopropyl dibenzyl phosphoramidite (1.6 g, 4.63 mmol) was stirred with 1*H*-tetrazole (0.45 mol/L in acetonitrile, 10.3 mL, 4.63 mol) in dry CH_2Cl_2 (20mL) under argon for 30min at room temperature. Compound **21** (1 g, 1.85mmol) dissolved in dry CH_2Cl_2 (30mL) was added

and the resulting mixture was stirred for 2h. TLC (petroleum ether/ethyl acetate 5:1) showed that the reaction was completed. The mixture was cooled to 0°C, and 3-chloroperoxybenzoic acid (800 mg, 4.63 mmol) was added. The resulting mixture was allowed to warm up to room temperature and stirred for 2h, the reaction was checked by TLC (petroleum ether/ethyl acetate 1:1). Then 10% aqueous solution of $NaHSO_4$ (2x100 mL) was added to quench m-CPBA, the layer was separated, and the aqueous layer was extracted with CH_2Cl_2 (2*100 mL). The combined organic layer were washed with HCl 1 M (2*100 mL), a saturated solution of $NaHCO_3$ (2x100 mL) and brine (2x100 mL) respectively. The organic phase was dried over $MgSO_4$, filtered, and concentrated under reduced pressure. Purification by silica gel column chromatography (petroleum ether/ethyl acetate 4:1) yield **22** (1.25 g, 84%) as a colorless oil. **^{1}H-NMR** (400 MHz, $CDCl_3$): 3.52 (dd, *J* = 2 and 10.8, 1H, H6), 3.59-3.66 (m, 2H, H6 and H4), 3.75-3.79 (m, 1H, H5), 3.97 (t, *J* = 9.3, 1H, H3), 4.30-4.35 (m, 1H, H2), 4.39-4.43 (m, 3H, CH_2Ph), 4.52-4.62 (m, 2H, CH_2Ph), 4.68-4.72 (m, 2H, CH_2Ph), 4.75-4.91 (m, 5H, CH_2Ph), 5.17 (d, *J* = 3.7, 1H, Hα), 7.02-7.05 (m, 2H, Ar), 7.07-7.10 (m, 2H, Ar), 7.12-7.26 (m, 26H, Ar); **^{13}C-NMR** (100 MHz, $CDCl_3$): 68.3 (C6), 69.2 (d, $J_{C\text{-}P}$ = 5.1, CH_2Ph), 69.4 (d, $J_{C\text{-}P}$ = 5.1, CH_2Ph), 70.0 (CH_2Ph), 70.6 (C5), 73.5 (CH_2Ph), 75.2 (CH_2Ph), 75.5 (CH_2Ph), 77.4 (d, $J_{C\text{-}P}$ = 5.9, C2), 77.8 (C4), 80.8 (d, $J_{C\text{-}P}$ = 7.3, C3) 96.6 (C1), 127.6, 127.8, 127.8, 127.8, 127.8, 127.9, 127.9, 128.0, 128.1, 128.4, 128.4, 128.4, 128.5, 128.5, 128.6, 135.9 (t, $J_{C\text{-}P}$ = 7.3, P-OCH_2-**C**Ar), 137.3, 138.0, 138.1, 138.5; **^{31}P-NMR**: (120 MHz, $CDCl_3$): -1.53. **$[\alpha]_D$** = +48.7 (*c*1.0, $CHCl_3$). **HRMS m/z (ESI):** Calcd. for $C_{48}H_{49}NaO_9P$ $(M+Na)^+$: 823.3006. Found: 823.2988.

D-Glucose-2-phosphate (**23**): Pd/C (40 mg) was added to a solution of compound **22** (0.1 g, 0.125 mmol) in methanol (10 mL) and the mixture stirred under an H_2 atmosphere for 18 h at room temperature. The solution was then filtered through celite and concentrated to give **23** (25 mg, 87%) as white solid. **^{1}H-NMR** (400 MHz, D_2O): 3.44-3.49 (m, 1.3H, H4α and β, H5β), 3.64 (t, *J* = 8.9, 0.4H, H3β), 3.67-3.90 (m, 3.85H, H2β, H3α, H5α, H6α and β), 3.94-4.0 (m, 0.7H, H2α), 4.74 (d, *J* = 7.7, 0.47H, Hβ), 5.39 (d, *J* = 3.2, 0.65H, Hα); **^{13}C-NMR** (100 MHz, D_2O): 61.1 (C6α), 61.3 (C6β), 69.9 (C4β), 70.0 (C4α), 71.9 (C5α), 72.1 ($J_{C\text{-}P}$ = 5.9, C3α), 75.6 ($J_{C\text{-}P}$ = 2.9, C3β), 76.2 ($J_{C\text{-}P}$ = 5.9, C2α), 76.5 (C5β), 79.5 ($J_{C\text{-}P}$ = 5.9, C2β), 91.3 (C1α), 95.5 ($J_{C\text{-}P}$ = 4.4, C1β) **^{31}P-NMR**: (200 MHz, D_2O): -0.26 (β) and -0.41 (α); **$[\alpha]_D$** = +40 (*c*1.0, MeOH). **HRMS m/z (ESI):** Calcd. for $C_6H_{12}O_9P$ $(M-H)^-$: 259.0224. Found: 259.0215.

Mass spectra:

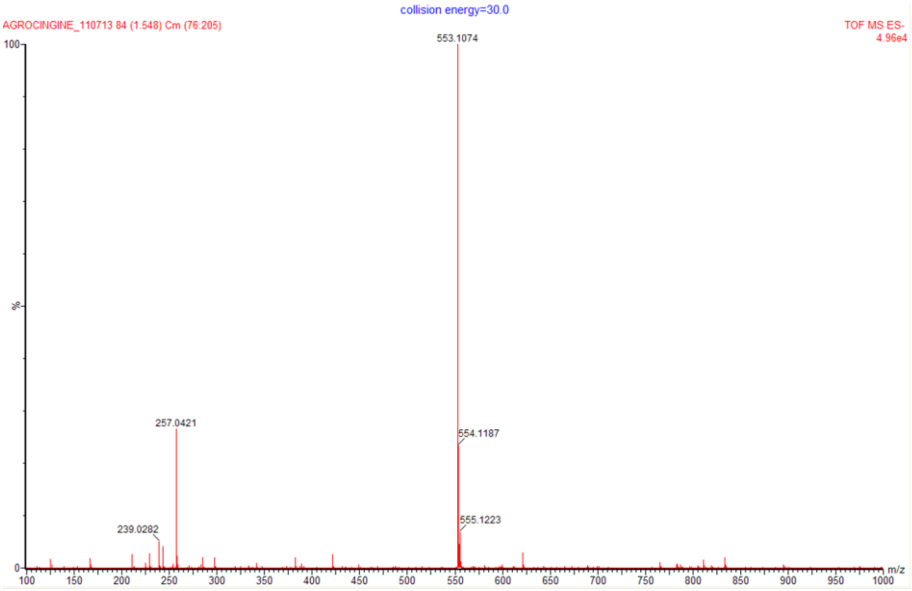

Agrocinopine A (MW 553 Da)

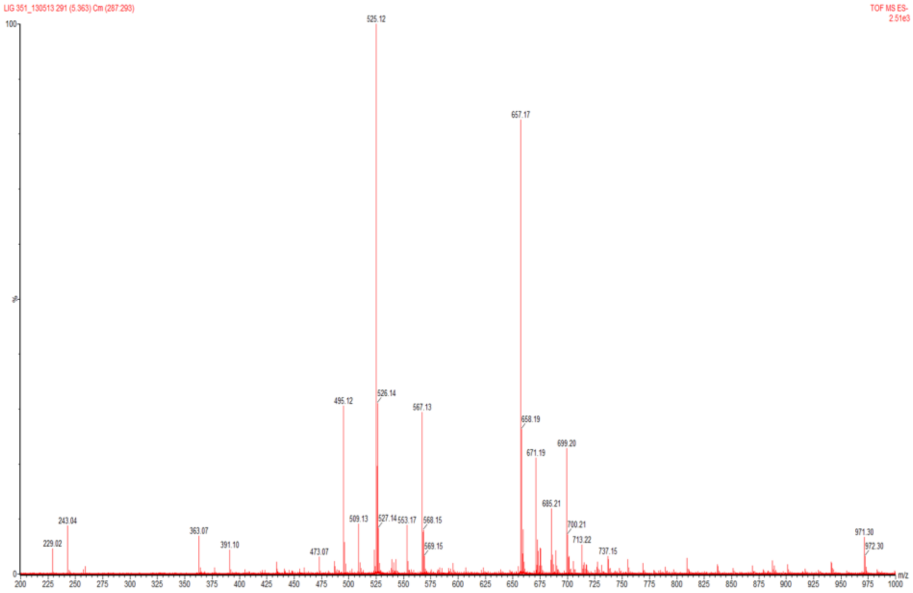

Agrocinopine 3’-*O*-benzoate (MW 657 Da)

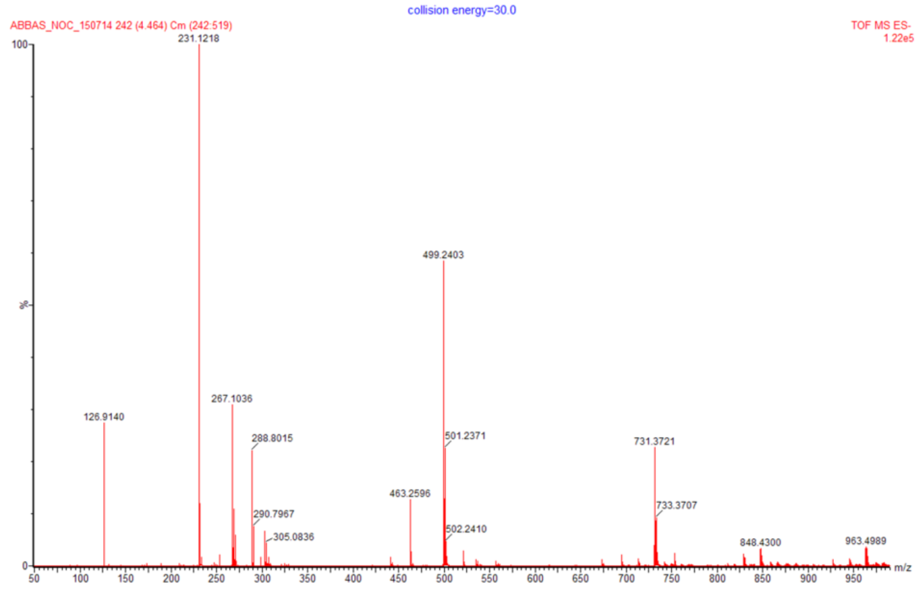

L-arabinose-2-phosphate (MW 231 Da)

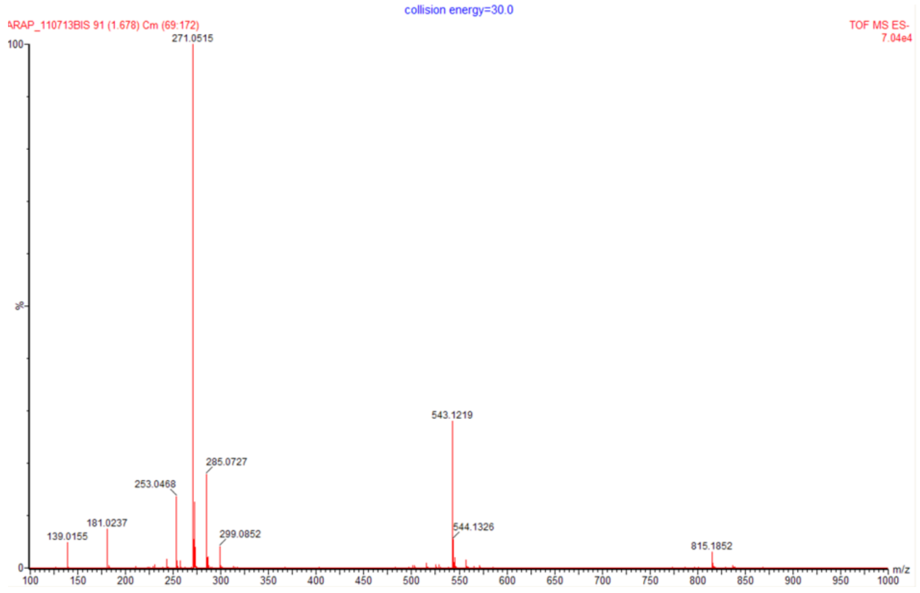

L-arabinose-2-isopropylphosphate (MW 271 Da)

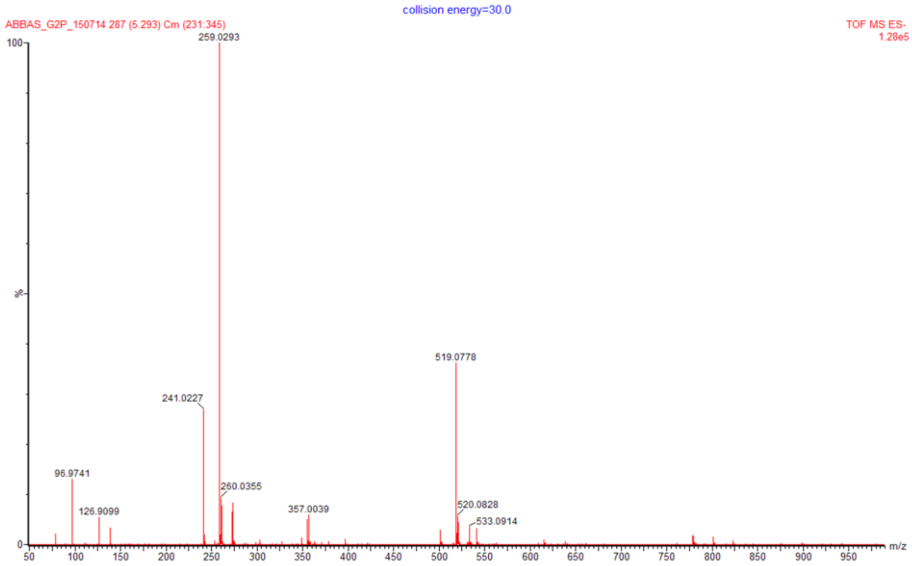

D-glucose-2-phosphate (MW 260 Da)

1. Hotha, S. & Kashyap, S. Propargyl glycosides as stable glycosyl donors: anomeric activation and glycoside syntheses. *J. Am. Chem. Soc.* **128,** 9620–1 (2006).

2. Suzuki, K., Nonaka, H. & Yamaura, M. Reductive Ring-Opening Reaction of 1,2- O -Benzylidene and 1,2- O - p -Methoxybenzylidene-α- D -glucopyranose Using Diisobutyl Aluminum Hydride. *J. Carbohydr. Chem.* **23,** 253–259 (2004).
